# Patient-reported outcomes of rezvilutamide versus bicalutamide in combination with androgen deprivation therapy in high-volume metastatic hormone-sensitive prostate cancer patients (CHART): a randomized, phase 3 study

**DOI:** 10.1038/s41392-024-02064-z

**Published:** 2024-12-18

**Authors:** Hongkai Wang, Shusuan Jiang, Hong Luo, Fangjian Zhou, Dalin He, Lulin Ma, Hongqian Guo, Chaozhao Liang, Tie Chong, Jun Jiang, Zhiwen Chen, Yong Wang, Qing Zou, Ye Tian, Jun Xiao, Jian Huang, Jinchao Chen, Qiang Dong, Xiaoping Zhang, Hanzhong Li, Xinfeng Yang, Jianpo Lian, Wenliang Wang, Dingwei Ye

**Affiliations:** 1 Department of Urology, Fudan University Shanghai Cancer Center, Fudan University, Shanghai, China; 2 Cancer Institute, Shanghai Urological Cancer Institute, Fudan University Shanghai Cancer Center, Fudan University, Shanghai, China; 3 Urology Surgery, Hunan Cancer Hospital, Changsha, China; 4 Department of Urology, Chongqing University Cancer Hospital, Chongqing, China; 5 Urology Surgery, Sun Yat-sen University Cancer Center, Guangzhou, China; 6 Urology Surgery, The First Affiliated Hospital of Xi’an Jiaotong University, Xi’an, China; 7 Urology Surgery, Peking University Third Hospital, Beijing, China; 8 Department of Urology, Nanjing Drum Tower Hospital, The Affiliated Hospital of Nanjing University Medical School, Nanjing, China; 9 Department of Urology, The First Affiliated Hospital of Anhui Medical University, Hefei, China; 10 Urology Surgery, The Second Affiliated Hospital of Xi’an Jiaotong University, Xi’an, China; 11 Department of Urology, Army Medical Center of PLA, Chongqing, China; 12 Department of Urology, The First Affiliated Hospital of the Army Medical University, Chongqing, China; 13 Department of Urology, The Second Hospital of Tianjin Medical University, Tianjin, China; 14 Department of Urology, Jiangsu Cancer Hospital, The Affiliated Cancer Hospital of Nanjing Medical University, Jiangsu Institute of Cancer Research, Nanjing, China; 15 Urology Surgery, Beijing Friendship Hospital Affiliated to Capital Medical University, Beijing, China; 16 Division of Life Sciences and Medicine, Department of Urology, The First Affiliated Hospital of USTC, University of Science and Technology of China, Hefei, China; 17 Urology Surgery, Sun Yat-sen Memorial Hospital, Sun Yat-sen University, Guangzhou, China; 18 Department of Urology, Zhejiang Cancer Hospital, Hangzhou, China; 19 Department of Urology, West China Hospital of Sichuan University, Chengdu, China; 20 Urology Surgery, Union Hospital, Tongji Medical College, Huazhong University of Science and Technology, Wuhan, China; 21 Urology Surgery, Peking Union Medical College Hospital, Beijing, China; 22 Department of Biometrics, Jiangsu Hengrui Pharmaceuticals Co. Ltd., Shanghai, China; 23 Clinical Research & Development, Jiangsu Hengrui Pharmaceuticals Co. Ltd., Shanghai, China

**Keywords:** Drug development, Drug development

## Abstract

The randomized phase 3 CHART trial (NCT03520478) revealed that rezvilutamide (REZ) plus androgen deprivation therapy (ADT) in high-volume, metastatic, hormone-sensitive prostate cancer (mHSPC) significantly enhanced radiographic progression-free and overall survival than bicalutamide (BIC)-ADT. Accordingly, we examined patient-reported outcomes (PROs) results, which were exploratory endpoints in the CHART trial. The patients were randomly allocated to receive REZ-ADT or BIC-ADT in a 1:1 ratio. The PROs were evaluated with the Brief Pain Inventory-Short Form (BPI-SF) and the Functional Assessment of Cancer Therapy-Prostate (FACT-P) questionnaires. Both study groups displayed comparable baseline pain scores and functional status. Patients administered REZ-ADT had an extended time to progression of worst pain intensity in comparison to those treated with BIC-ADT (25th percentile, 9.2 [95% CI 7.4–16.6] vs. 6.4 months [95% CI 5.5–8.3]; HR 0.75 [95% CI 0.57–0.97]; *p* = 0.026). Similarly, patients received REZ-ADT exhibited a delayed time to progression of pain interference in comparison to those receiving BIC-ADT (25th percentile, 20.2 [95% CI 12.9–31.3] vs. 10.2 months [95% CI 7.4–11.1]; HR 0.70 [95% CI 0.52–0.93]; *p* = 0.015). Additionally, the REZ-ADT group demonstrated a prolonged delay in the deterioration of the total score on the FACT-P questionnaire (25th percentile, 12.8 [95% CI 7.4–20.3] vs. 6.0 months [95% CI 4.6–9.2]; HR 0.66 [95% CI 0.50–0.86]; *p* = 0.002), as well as most of the FACT-P subscale scores, in comparison to the BIC-ADT group. In conclusion, REZ-ADT is superior to BIC-ADT regarding the pain alleviation and enhancement of functional scales for high-volume mHSPC.

## Introduction

Prostate cancer is among the prevailing reasons for cancer-linked morbidity and death in men worldwide, with its incidence rising as populations age.^1^ Metastatic hormone-sensitive prostate cancer (mHSPC) is defined by a particularly aggressive disease type, where the tumor spreads beyond the prostate gland, commonly to the bones and lymph nodes, leading to challenging complications and reduced survival. Patients with mHSPC experience a high morbidity rate and poor clinical outcomes, and this disease severely impacts their quality of life (QoL), with common symptoms including bone pain, fatigue, urinary issues, and psychosocial distress.^2^ This underscores the need for developing treatment approaches that not only effectively control disease progression but also enhance or maintain patient-reported outcomes (PROs) in this population.

The androgen receptor axis is crucial in prostate cancer pathology by driving tumor proliferation and growth.^3,4^ While androgen-deprivation therapy (ADT), which reduces circulating testosterone levels, has been the cornerstone of treatment, single-agent ADT alone often fails to sustain long-term efficacy, as prostate cancer cells develop adaptive resistance to testosterone suppression. In recent years, the emergence of androgen receptor axis inhibitors, encompassing enzalutamide, abiraterone acetate, and apalutamide, has achieved a significant advancement in the therapeutic landscape for mHSPC. These agents, when used in combination with ADT, have demonstrated considerable survival benefits and have become integral to the standard of care for mHSPC patients.^5–8^ The combination of androgen receptor axis inhibitors with ADT more effectively suppresses androgen receptor signaling than ADT alone, offering new hope for delaying disease progression and improving survival outcomes in mHSPC patients.^9^ For high-volume mHSPC patients who exhibit an extensive tumor burden with multiple metastatic sites, these new drugs have shown potential in clinical trials, resulting in prolonged progression-free survival (PFS) and overall survival (OS) benefits. Notwithstanding these advancements, some patients experience resistance to existing androgen receptor-targeting therapies,^9^ underscoring the need for novel agents that can further enhance clinical outcomes, especially for high-volume cases where the tumor burden is substantial and aggressive. Consequently, researchers have been investigating new-generation androgen receptor inhibitors that may overcome treatment resistance, improve efficacy, reduce toxicity, and maintain or enhance QoL, which are essential needs in managing the disease course of mHSPC patients.

Rezvilutamide (REZ) is a new orally administrated inhibitor of the androgen receptor that specifically targets the androgen receptor axis.^10,11^ A preclinical study conducted in mice has indicated that REZ penetrates the blood-brain barrier to a lesser extent compared to enzalutamide, potentially lowering the seizure risk. In metastatic castration-resistant prostate cancer patients, clinical studies have demonstrated the promising efficacy and favorable safety profile of REZ.^12,13^ The CHART trial, a randomized phase 3 study, was performed to ascertain the efficacy and safety of REZ-ADT in high-volume mHSPC patients compared to bicalutamide (BIC)-ADT. This study manifested that the REZ-ADT significantly enhanced OS (Hazard ratio [HR] 0.58 with 95% confidence interval [CI] 0.44–0.77; p = 0.0001) and radiographic progression-free survival (rPFS; HR 0.44 [95% CI 0.33–0.58]; p < 0.0001) in mHSPC patients when compared with BIC-ADT.^10,14^ These outcomes resulted in the approval of REZ-ADT in China in 2022 for treating high-volume mHSPC.^11^ The CHART trial results underscore the importance of incorporating novel androgen receptor-targeting agents into the treatment paradigm for metastatic prostate cancer, especially given the growing body of evidence that these therapies can enhance clinical outcomes and offer new hope for patients with poor prognosis.

In addition to improving clinical efficacy, PROs have emerged as critical study endpoints in the evaluation of treatment approaches for mHSPC. Patients with mHSPC often endure not only the physical burden of the disease but also a spectrum of adverse effects from treatment, such as fatigue, hot flashes, loss of libido, and cognitive changes.^15,16^ These side effects, combined with disease-related symptoms, can severely impact the QoL and mental well-being of patients.^17^ Furthermore, mHSPC disproportionately affects older adults, who may have additional comorbidities that can exacerbate the impact of both disease symptoms and treatment toxicity. Traditional clinical study endpoints, such as OS and rPFS, while essential for assessing treatment efficacy, often fail to capture the daily QoL concerns of patients.^18^ Moreover, PROs, which assess symptom burden, functional status, and overall satisfaction with therapy from the perspectives of patients, offer valuable insights into how treatments impact daily functioning and overall well-being, providing a more comprehensive view of therapeutic benefits.^19,20^ Incorporating PROs into the study endpoints of clinical trials conducted in mHSPC patients allows for patient-centered evaluations and informs decision-making by simultaneously considering efficacy, safety, and QoL, ultimately aiming to optimize comprehensive care and improve patient satisfaction.^18^

In the CHART trial, PROs were assessed as exploratory endpoints, focusing on pain control and functional assessments in mHSPC patients receiving either REZ-ADT or BIC-ADT. In this report, we present the comparative PRO data from the CHART phase 3 trial, underscoring the importance of evaluating patient-centered outcomes alongside efficacy and safety in treatment strategies for high-volume mHSPC.

## Results

### Patients

Between June 28, 2018, and August 6, 2020, 654 patients fulfilling the eligibility criteria were recruited, with 326 and 328 patients allocated to the REZ-ADT and BIC-ADT groups, respectively. Baseline characteristics, including demographics, disease status, prior treatments, pain levels, and functional scores, were comparable across both treatment groups (Table 1).^14^

**Table 1:** Baseline characteristics and PRO scores

	Rezvilutamide plus ADT (*n* = 326)	Bicalutamide plus ADT (*n* = 328)
Age, years, median (IQR)	69 (64–74)	69 (64–75)
Gleason score, *n* (%)		
<8	47 (14%)	64 (20%)
≥8	276 (85%)	257 (78%)
Missing data	3 (1%)	7 (2%)
Extent of metastatic disease, *n* (%)		
Bone only	123 (38%)	127 (39%)
Soft tissue only	8 (2%)	2 (1%)
Bone and soft tissue	195 (60%)	199 (61%)
Baseline BFI-SF scores, mean (SD)		
Worst pain in the past 24 h	1.5 (2.37)	1.6 (2.56)
Pain interference	0.9 (1.98)	1.0 (1.97)
Average pain	1.0 (1.63)	1.0 (1.63)
Pain severity per score, *n* (%)		
No pain (0)	203 (62%)	206 (63%)
Mild (1–3)	54 (17%)	50 (15%)
Moderate (4–7)	62 (19%)	58 (18%)
Severe (8–10)	7 (2%)	14 (4%)
Baseline FACT-P scores, mean (SD)		
FACT-P total scale	121.1 (19.42)	121.4 (20.44)
Physical well-being	23.5 (4.05)	23.5 (4.39)
Social/family well-being	23.8 (4.40)	24.0 (4.27)
Emotional well-being	20.4 (3.80)	20.5 (3.82)
Functional well-being	19.1 (6.18)	19.3 (6.11)
FACT-G general scale	86.8 (13.77)	87.2 (14.09)
Prostate cancer subscale	34.3 (7.28)	34.2 (7.99)
Trial outcome index	76.8 (14.70)	76.9 (15.69)
FACT-P pain scale	12.6 (3.81)	12.5 (4.09)

This exploratory analysis of PROs had a median follow-up period of 29.3 months (interquartile range [IQR]: 21.0–33.3), with a data cutoff date of February 28, 2022. The PRO data beyond 44 treatment cycles were excluded because the BIC-ADT group had a limited number of remaining patients.

### Brief Pain Inventory-Short Form (BPI-SF)

Compliance rates for BPI-SF were high throughout the study, with over 90% compliance observed in both treatment groups up to week 161 (Supplementary Table 1). Only 25 patients (3.8%) missed three or more scheduled pain assessments. Baseline assessments showed low pain levels across both groups, with 203 (62%) in the REZ-ADT group and 206 (63%) in the BIC-ADT group reporting no pain at baseline, while 17% and 15%, respectively, reported mild pain (Table 1). Notably, Asian patients generally reported lower baseline pain levels than non-Asian patients (Supplementary Table 2).

**Fig. 1:**
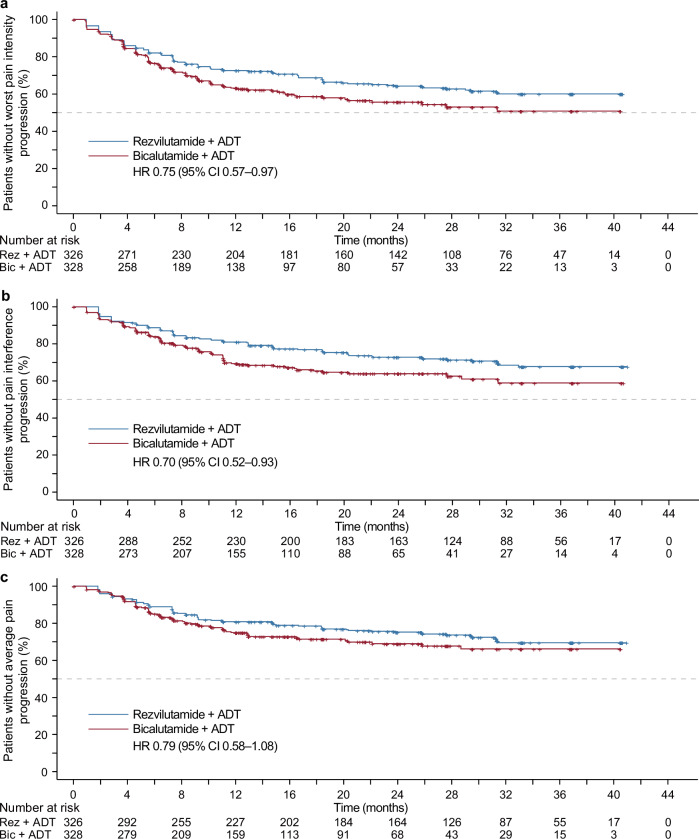
Kaplan–Meier curves for time to pain progression assessed by BPI-SF. **a** time to worst pain progression. **b** time to pain interference progression. **c** time to average pain progression

Patients treated with REZ-ADT demonstrated an extended time to progression of worst pain intensity, in contrast to those receiving BIC-ADT (median: NR [95% CI NR–NR] vs. NR [95% CI 20.3–NR]; 25th percentile: 9.2 [95% CI 7.4–16.6] vs. 6.4 months [95% CI 5.5–8.3]; HR 0.75 [95% CI 0.57–0.97]; *p* = 0.026, Fig. 1a). Similarly, the REZ-ADT group experienced an extended time to progression in pain interference (median: NR [95% CI NR–NR] vs. NR [95% CI NR–NR]; 25th percentile: 20.2 [95% CI 12.9–31.3] vs. 10.2 months [95% CI 7.4–11.1]; HR 0.70 [95% CI 0.52–0.93]; *p* = 0.015, Fig. 1b). Both groups did not achieve the median time to average pain progression; the REZ-ADT group showed 25th percentile values of 25.8 months (95% CI 14.8–31.4), while the BIC-ADT group indicated 11.7 months (95% CI 8.7–22.1; HR 0.79 [95% CI 0.58–1.08]; *p* = 0.133; Fig. 1c).

**Fig. 2:**
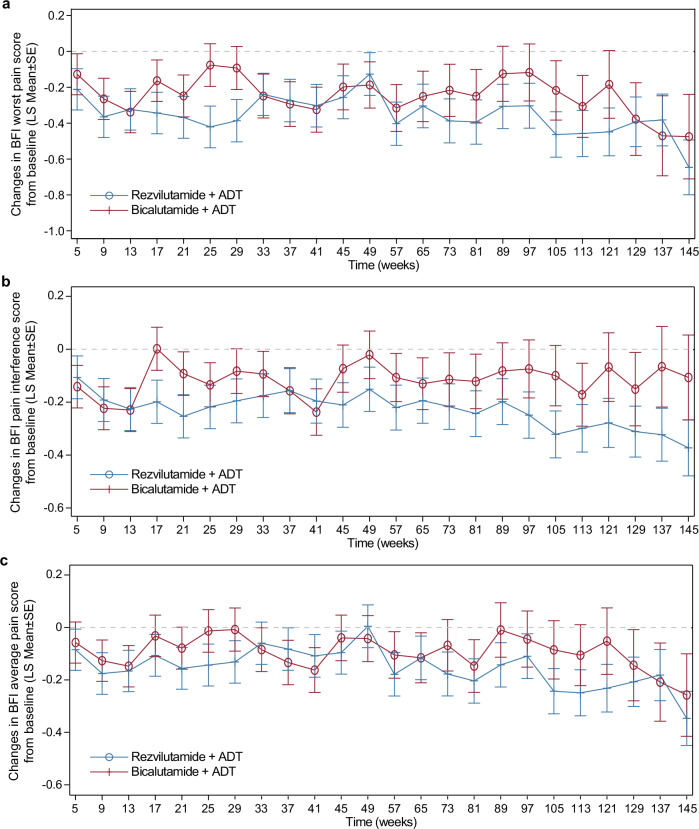
Changes in pain from baseline assessed by BPI-SF. **a** Worst pain in past 24 h. **b** Pain interference. **c** Average pain

Both treatment groups exhibited decreases in worst pain intensity, interference, and average pain over time, with more pronounced reductions found in the REZ-ADT group at various time intervals (Fig. 2). Analysis of the initial 12 treatment cycles stratified by baseline pain severity revealed that patients reporting moderate baseline pain showed marked improvements across pain metrics, with least-squares mean (LS Mean) reductions ranging from –1.96 to –3.26 for worst pain intensity score, –0.54 to –1.78 for pain interference score, and –1.00 to –1.97 for average pain score in both groups. The REZ-ADT group frequently experienced a greater enhancement of pain relief than the BIC-ADT cohort (Supplementary Figs. 1–3). Meanwhile, patients with no or mild baseline pain maintained stable pain levels throughout the study.

Baseline assessments indicated higher PSA levels and a greater number of bone metastatic lesions among patients experiencing moderate to severe baseline pain compared to those with mild or no pain in both the REZ-ADT and BIC-ADT groups (Supplementary Table 3). Moreover, no obvious correlations were found between pain severity at baseline and the presence of visceral metastases. Across all baseline pain levels, the REZ-ADT combination consistently delayed PSA progression and prolonged rPFS and OS compared to BIC-ADT, with HRs < 1 across most efficacy outcomes, except for OS improvement in the subgroup with severe pain (Supplementary Table 4). This suggests a consistent therapeutic advantage of REZ-ADT, regardless of initial pain severity.

Additional analyses were conducted to examine the efficacy of REZ-ADT compared to BIC-ADT in delaying the progression of worst pain intensity, pain interference, and average pain in Asian and non-Asian patient subgroups (Supplementary Table 5). Due to a smaller sample size of non-Asian patients, findings for this subgroup should be interpreted with caution.

### Functional Assessment of Cancer Therapy-Prostate (FACT-P)

The overall compliance with the FACT-P questionnaire was strong, with adherence rates above 90% in both treatment groups through week 161 (Supplementary Table 1). Only 25 patients (3.8%) missed three or more scheduled functional assessments. Baseline FACT-P scores indicated that the functional status was similar between the REZ-ADT and BIC-ADT groups (Table 1).

**Fig. 3:**
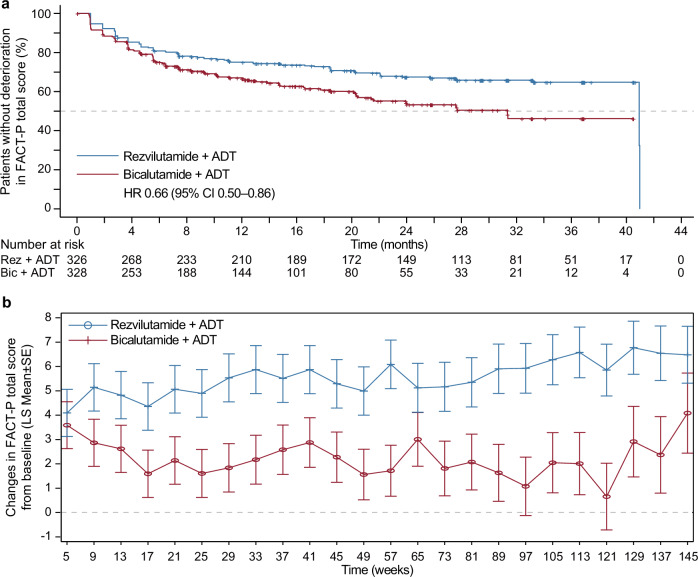
FACT-P total score. **a** Kaplan–Meier curve for time to functional status deterioration assessed by FACT-P total score. **b** Change from baseline in FACT-P total score

For the FACT-P total score, REZ-ADT had longer 25th percentile time to functional deterioration (12.8 months [95% CI 7.4–20.3] compared to 6.0 months [95% CI 4.6–9.2] with BIC-ADT; HR 0.66 [95% CI 0.50–0.86]; *p* = 0.002; Fig. 3a, Table 2). Deterioration across nearly all FACT-P subscales was delayed with the administration of REZ-ADT, encompassing delayed deterioration in physical well-being (HR 0.65, 95% CI 0.49–0.86; *p* = 0.003), emotional well-being (HR 0.68, 95% CI 0.50–0.92; *p* = 0.013), functional well-being (HR 0.75, 95% CI 0.59–0.95; *p* = 0.015), the FACT-G general scale (HR 0.69, 95% CI 0.52–0.91; *p* = 0.008), the prostate cancer subscale (HR 0.74, 95% CI 0.57–0.96; *p* = 0.022), and the trial outcome index (HR 0.65, 95% CI 0.49–0.86; *p* = 0.002**;** Table 2). Nonetheless, no difference was found between the groups for the time to deterioration in social/family well-being or the FACT-P pain scale.

**Table 2:** Time to deterioration of functional status in FACT-P total scale and subscales

	25th percentile (95% CI)^a^, months		HR (95% CI)	*P* value
	Rezvilutamide plus ADT (*n* = 326)	Bicalutamide plus ADT (*n* = 328)		
FACT-P total scale	12.8 (7.4–20.3)	6.0 (4.6–9.2)	0.66 (0.50–0.86)	0.002
Physical well-being	18.4 (8.3–36.8)	7.4 (5.6–11.1)	0.65 (0.49–0.86)	0.003
Social/family well-being	4.6 (3.6–6.5)	4.6 (3.7–5.6)	0.89 (0.70–1.13)	0.332
Emotional well-being	29.0 (14.9–NR)	12.4 (8.3–18.4)	0.68 (0.50–0.92)	0.013
Functional well-being	4.7 (3.7–6.5)	4.2 (2.8–5.5)	0.75 (0.59–0.95)	0.015
FACT-G general scale	12.8 (7.4–23.9)	8.4 (5.6–12.6)	0.69 (0.52–0.91)	0.008
Prostate cancer subscale	9.2 (6.5–13.8)	5.5 (3.8–7.3)	0.74 (0.57–0.96)	0.022
Trial outcome index	14.8 (7.4–27.6)	8.2 (5.7–11.1)	0.65 (0.49–0.86)	0.002
FACT-P pain scale	5.6 (3.7–7.4)	4.6 (3.7–6.2)	0.85 (0.67–1.08)	0.184

^a^The 25th percentiles were reported here because the majority of the median values had not been reached

Both groups demonstrated improvements from baseline in the FACT-P total score at all time points (LS Means of 0.66**–**6.77; Fig. 3b), with the REZ-ADT group showing more pronounced improvements, reflected in between-group LS Mean differences of 0.50–5.20. The score changes in all FACT-P subscales from baseline exhibited similar patterns in the improvement in both groups, along with between-group differences, except in the subscales of social/family well-being and the FACT-P pain scale.

An additional analysis evaluated the relationship between FACT-P total score changes over the initial 12 treatment cycles and baseline pain levels. Results suggested that patients with higher baseline pain reported greater improvements in the FACT-P total scale. While patients with low to no pain at baseline showed minor improvements, those with moderate baseline pain reported substantial improvements, with LS Mean scores of 0.92–12.16 overall assessment time points. This trend was more pronounced in the REZ-ADT group, consistently exceeding improvements in the BIC-ADT group, with between-group LS Mean differences of 3.70–8.57 (Supplementary Fig. 4).

## Discussion

This exploratory analysis of the phase 3 CHART study indicates that REZ-ADT outperformed BIC-ADT in high-volume mHSPC patients in terms of delaying pain progression and enhancing functional health. Treatment with REZ-ADT demonstrated a delayed progression in worst pain intensity, pain interference, and functional deterioration, as assessed by BPI-SF and FACT-P, respectively. The high patient adherence observed for PRO measures supports the reliability of these assessments in capturing clinically relevant improvements with REZ-based therapy.

Pain management is fundamental in mHSPC care due to its direct impact on patient QoL.^21,22^ Our findings suggest that the REZ-ADT group experienced an extension in time to pain progression—specifically, in terms of both worst pain and interference scores compared to the BIC-ADT group. However, differences in average pain progression time between groups were minimal. These results align with findings from the LATITUDE trial, which demonstrated that ADT-abiraterone acetate and prednisone delayed the progression of worst pain intensity and interference compared to ADT-placebos.^23^ Conversely, the TITAN and ARCHES studies demonstrated no notable differences between apalutamide-ADT and placebo-ADT or enzalutamide-ADT and placebo-ADT regarding the time to progression of worst pain intensity, pain interference, and average pain progression.^24,25^ Variations in trial design and patient features should be considered when comparing these results, underscoring the need for further studies on androgen receptor inhibitors combined with ADT to better understand their effects on pain progression.

The analysis of pain relief from baseline scores across worst pain intensity and interference, and average pain favored REZ-ADT at most assessed intervals, suggesting a stronger and more sustained pain-relief effect in this treatment group. Particularly, patients with greater initial pain severity exhibited more substantial improvements in pain-related measures when treated with REZ-ADT, indicating a potential predictive value of baseline pain levels for clinical response to this therapy. These findings are consistent with data from the TITAN study, where apalutamide-ADT was also more effective in patients with higher baseline pain severity.^24^

Previous studies have shown that there can be variations in pain reporting across ethnic groups, potentially influenced by both cultural and biological factors. Consistent with earlier findings, our results indicated that Asian patients reported lower baseline pain levels,^26^ possibly reflecting cultural tendencies such as stoicism, which may affect pain reporting. In contrast, studies suggested that Black men may report higher pain levels than Caucasians, possibly due to variations in pain tolerance, access to healthcare, and historical disparities in pain management for minority populations.^26^ From a biological perspective, differences in androgen receptor gene polymorphisms, tumor pathobiology, and hormone metabolism have been observed across ethnic groups,^26^ may also contribute to these observed differences in pain reporting both at baseline and during the androgen receptor inhibitor treatment. Although the CHART trial did not specifically address these factors, understanding these variations is essential for interpreting treatment outcomes in diverse patient groups. Future studies in more demographically varied populations, with subgroup analyses by ethnicity, would provide valuable perspectives into the efficacy of REZ-ADT for managing pain in mHSPC across different backgrounds.

Our analysis using the FACT-P questionnaire revealed that patients treated with REZ-ADT experienced a longer time to functional deterioration across the total scale and more subscales than those in the BIC-ADT group, indicating a sustained preservation of functional well-being. This result suggests that REZ-ADT supports a better QoL by delaying declines in overall functioning. Although prior studies have shown positive impacts of androgen receptor inhibitors combined with ADT on functional outcomes,^23–25^ our findings add robust evidence to this growing body of research. However, differences between groups in social/family well-being and the FACT-P pain scale were not obvious, warranting further exploration to understand the underlying causes.

The trend of improvement in FACT-P scores from baseline across the total scale and nearly all subscales consistently favored REZ-ADT, underscoring its efficacy in sustaining functional well-being in high-volume mHSPC patients. Correlation analyses showed that patients with higher baseline pain severity experienced the most notable improvements, suggesting that baseline characteristics like pain severity could guide more individualized treatment strategies.

The progression of pain, PSA progression, and survival outcomes relative to metastatic burden observed in the BIC arm were generally consistent with existing findings,^7,14,27–31^ supporting the representativeness of this study population and the reliability of the findings. Although direct comparisons across studies should be interpreted cautiously, the data here suggest a distinct advantage in both efficacy and PROs for REZ over BIC when used with ADT in high-volume mHSPC. The observed improvement in survival and maintenance or enhancement of QoL in patients treated with REZ-ADT can likely be attributed to several potential mechanisms. The selective inhibition of REZ on androgen receptor pathways likely plays a central role by blocking cancer cell growth, reducing tumor burden, and delaying disease progression, especially pronounced in bone metastases where pain and functional decline are common.^14^ Additionally, REZ may help maintain physical mobility and reduce discomfort linked to bone metastasis by limiting osteoclast-mediated bone breakdown. This inhibition of bone resorption could alleviate complications and support mobility, addressing an important QoL concern for high bone metastatic burden patients.^32,33^ Another possible mechanism involves the influence of REZ on molecules associated with pain, such as nerve growth factor and inflammatory cytokines at metastatic sites,^34^ which could lead to pain reduction. Moreover, its regulatory impact on the hypothalamic-pituitary-adrenal axis,^35,36^ may reduce stress hormone levels, such as cortisol, contributing to improved mental well-being and, in turn, better physical functioning. Altogether, these mechanisms underscore that the survival benefits of REZ-ADT do not come at the expense of QoL; instead, they may enhance it. These insights enable clinicians to have more comprehensive discussions with patients, offering a treatment choice that prioritizes both extended survival and sustained QoL.

Herein, there are several limitations. The current follow-up duration of 29.3 months may not fully capture the entire spectrum of PROs, particularly in the REZ-ADT group, which exhibited superior anti-tumor efficacy and a more favorable PRO profile compared to the BIC-ADT group, with a substantial proportion of patients still receiving treatment. Therefore, an extended follow-up period will be crucial for assessing the long-term durability of these findings. Additionally, since most participants were Asian, the applicability of our study to broader, ethnically diverse populations might be limited. Future studies and meta-analyses incorporating global data can help assess the consistency of these results across different demographics, enhancing our understanding of the broader applicability of REZ-ADT for high-volume mHSPC patients worldwide.

To conclude, the CHART study establishes the benefits of REZ over BIC when combined with ADT for managing pain and maintaining functional status in high-volume mHSPC. Together with its demonstrated anti-tumor efficacy and safety, these findings reinforce REZ-ADT as a promising treatment option and a potential new standard of therapy for these patients.

## Methods

### Study design and patient population

In 72 hospitals across China, Poland, the Czech Republic, and Bulgaria, a multinational, randomized, open-label, active-controlled phase 3 CHART study (NCT03520478) was performed. Eligibility criteria were men aged 18 years or above, having histologically or cytologically verified high-volume prostate adenocarcinoma. High-volume disease was characterized by the existence of four or more bone lesions identified using a [99Tc] bone scan, with a minimum of one lesion situated outside the pelvis or vertebral column or by the presence of visceral metastases (excluding lymph node involvement) verified via CT or MRI. Eligible patients needed to have an Eastern Cooperative Oncology Group (ECOG) performance status of 0 or 1 and sufficient organ function. Prior treatment with ADT was permitted if administered no more than three months before study entry, provided there was no evidence of radiographic or clinical progression of prostate-specific antigen levels. Patients exhibiting neuroendocrine differentiation or small-cell characteristics, or those with a history of chemotherapy or localized treatment, were excluded. Nevertheless, patients who had received a single course of palliative radiotherapy, transurethral resection of the prostate, or surgical interventions for metastatic symptoms were eligible, provided these interventions were completed at least four weeks prior to treatment initiation and all treatment-related adverse events resolved to grade 1 or 0, as per the Common Terminology Criteria for Adverse Events version 4.03.

The study protocol and its amendments were approved by independent ethics review committee at each involved site. The trial adhered to the Declaration of Helsinki and the International Council for Harmonization Good Clinical Practice guidelines. All participants provided informed consent.

### Treatment and assessments

Eligible patients were randomly allocated to receive ADT (utilizing a luteinizing hormone-releasing hormone agonist or antagonist or bilateral orchiectomy) combined with either REZ (240 mg) or BIC (50 mg) in a 1:1 ratio, received orally once daily in 28-day therapy cycles. Randomization was stratified according to ECOG performance status (0 or 1) and the existence of visceral metastases (yes or no). Treatment continued until disease progression, unacceptable toxicity, withdrawal of consent, or decision by the investigator. Dose interruptions and reductions for REZ were allowed, but dose interruptions for BIC were allowed to manage toxicity, but dose reductions were not permitted. Pain management was guided by the World Health Organization analgesic ladder and local pain management protocols in China.^37–40^

The PROs were evaluated with BPI-SF and the FACT-P version 4 questionnaires. The BPI-SF has 15 items that evaluate two main domains: pain severity and pain interference.^41,42^ Pain severity and its effect on everyday activities were rated on a scale from 0 to 10, with 0 denoting “no pain” or “no interference” and 10 signifying “the worst imaginable pain” or “complete interference.” Higher scores indicate worse pain experiences. The FACT-P questionnaire encompasses domains of physical, social, family, emotional, and functional well-being, and a prostate cancer-specific domain. It comprises the total FACT-P scale (0–156), the FACT-P subscale for general functional status (FACT-G, which includes physical, social and family, emotional, and functional well-being; 0–108), and the trial outcome index (which includes physical and functional well-being, and the prostate cancer-specific domain), with higher scores reflecting better outcomes.^43–45^ At baseline, the questionnaires were administered, as well as at the commencement of each cycle from cycles 2 to 12, bi-cyclically during cycles 13 to 36, and every four cycles afterward, continuing until 30 days following the last treatment. Patient-reported data were not collected throughout long-term survival follow-ups.

### Outcomes

The co-primary endpoints of the CHART trial encompassed independent review committee-evaluated rPFS and OS, with data previously published. PROs were exploratory endpoints evaluated using the BPI-SF and the FACT-P (version 4).

The time to the worst pain intensity progression was defined as the interval from randomization to the first occurrence of a 2-point or greater rise in pain intensity, as assessed by item 3 of the BPI-SF. The time to pain interference progression was calculated as the period from randomization to the first occurrence of an increase of at least half a standard deviation from baseline scores on the combined scale of items 9A-G of the BPI-SF. The time to average pain progression was defined as the period from randomization to the first reported rise of 2 points or more in average pain compared to baseline, determined by the average of items 3–6 of the BPI-SF. Deterioration thresholds for the FACT-G general scale, trial outcome index, FACT-P total scale, and FACT-P pain scale were established at 9 points, 9 points, 10 points, and 2 points, respectively; for other FACT-P scales; the threshold for deterioration was set at 3 points. Confirmatory evaluations for these progressions were required at subsequent assessments conducted at least four weeks later. In cases where the last assessment indicated progression but there was no confirmed progression, the last assessment was treated as an event.

### Statistical analyses

Details regarding sample size assumptions have been previously reported. PROs were analyzed in the intent-to-treat population, including all randomized patients.

Compliance, BPI-SF scale scores, and FACT-P scale scores were summarized descriptively. The Kaplan–Meier methodology was employed to calculate the median time to deterioration for each therapy group, with 95% CIs for median values estimated with the Brookmeyer-Crowley method. Between-group differences in time-to-event parameters were analyzed utilizing a stratified log-rank test. A stratified Cox proportional hazards model was emloyed to derive the HR and their corresponding 95% CIs. Between-group comparisons of PSA progression, rPFS, and OS across different baseline pain severity levels, as well as between-group comparisons of pain progression between Asians and non-Asians, were not stratified. In cases where median values could not be ascertained, comparisons were made using the 25th percentiles. The censoring date for time to PRO progression or deterioration of the BPI-SF and FACT-P was defined as the date of the last BPI-SF assessment if no pain progression was observed or the date of randomization if there was no baseline or post-baseline disease assessment.

Changes from baseline in the BPI-SF and FACT-P scales were analyzed employing a linear Mixed Model Repeated Measures model approach, employing Restricted Maximum Likelihood estimation. The model incorporated the baseline values, treatment groups, stratification factors, visits, and treatment-by-visit interactions, treating patients as random effects. An unstructured covariance matrix was used, with a fallback to compound symmetry covariance structures if convergence issues arose. The Kenward-Roger approximation was employed to investigate the freedom degrees. The LS Mean and the differences between groups in LS Means, along with their corresponding 95% CIs, were calculated. The model included visits with at least 30 non-missing values in each treatment group. All statistical analyses were conducted utilizing SAS software (v. 9.4).

## Supplementary information

coi_disclosure

Supplementary Tables and Figures

Plagiarism checking report

CONSORT Checklist

## Data Availability

Available upon a reasonable request from the corresponding author.
