# Supplementary material for: Patient-reported outcomes of rezvilutamide versus bicalutamide in combination with androgen deprivation therapy in high-volume metastatic hormone-sensitive prostate cancer patients (CHART): a randomized, phase 3 study
Source: Signal Transduct Target Ther. 2024 Dec 18;9:351. doi: 10.1038/s41392-024-02064-z (PMC11652652; doi:10.1038/s41392-024-02064-z)
Supplement: Supplementary file 2 — Supplementary Tables and Figures [file 41392_2024_2064_MOESM2_ESM.docx]

Supplementary Materials for

Patient-reported outcomes of rezvilutamide versus bicalutamide in combination with androgen deprivation therapy in high-volume metastatic hormone-sensitive prostate cancer patients (CHART): a randomized, phase 3 study

Hongkai Wang, Shusuan Jiang, Hong Luo, Fangjian Zhou, Dalin He, Lulin Ma, Hongqian Guo, Chaozhao Liang, Tie Chong, Jun Jiang, Zhiwen Chen, Yong Wang, Qing Zou, Ye Tia, Jun Xiao, Jian Huang, Jinchao Che, Qiang Dong, Xiaoping Zhang, Hanzhong Li, Xinfeng Yang, Jianpo Lian, Wenliang Wang, Dingwei Ye

Correspondence to: dwyeli@163.com

**This PDF file includes:**

Tables S1 to S5

Figures S1 to S4

## Table S1. Compliance rate

|  | **BPI-SF** | | **FACT-P** | |
| --- | --- | --- | --- | --- |
|  | **Rezvilutamide plus ADT** | **Bicalutamide plus ADT** | **Rezvilutamide plus ADT** | **Bicalutamide plus ADT** |
| Baseline | 99.7 (325/326) | 100 (328/328) | 100 (326/326) | 100 (328/328) |
| Week 5 | 99.1 (319/322) | 99.4 (318/320) | 99.1 (319/322) | 99.4 (318/320) |
| Week 9 | 96.9 (312/322) | 97.2 (308/317) | 96.9 (312/322) | 97.2 (308/317) |
| Week 13 | 98.1 (315/321) | 96.2 (305/317) | 98.4 (316/321) | 96.2 (305/317) |
| Week 17 | 97.5 (307/315) | 96.5 (303/314) | 97.8 (308/315) | 96.5 (303/314) |
| Week 21 | 98.1 (305/311) | 97.0 (290/299) | 98.1 (305/311) | 97.0 (290/299) |
| Week 25 | 97.7 (301/308) | 95.8 (272/284) | 97.4 (300/308) | 96.1 (273/284) |
| Week 29 | 94.7 (284/300) | 94.9 (258/272) | 94.7 (284/300) | 94.9 (258/272) |
| Week 33 | 96.0 (287/299) | 94.2 (242/257) | 96.0 (287/299) | 94.2 (242/257) |
| Week 37 | 96.2 (279/290) | 94.1 (225/239) | 96.2 (279/290) | 94.6 (226/239) |
| Week 41 | 96.5 (276/286) | 96.1 (219/228) | 96.9 (277/286) | 96.1 (219/228) |
| Week 45 | 94.0 (265/282) | 94.9 (205/216) | 94.3 (266/282) | 94.9 (205/216) |
| Week 49 | 97.5 (272/279) | 95.2 (198/208) | 97.8 (273/279) | 95.2 (198/208) |
| Week 57 | 94.8 (257/271) | 97.9 (189/193) | 94.8 (257/271) | 97.9 (189/193) |
| Week 65 | 95.0 (249/262) | 91.2 (145/159) | 95.0 (249/262) | 91.2 (145/159) |
| Week 73 | 96.8 (242/250) | 91.7 (132/144) | 96.8 (242/250) | 91.7 (133/145) |
| Week 81 | 96.3 (235/244) | 93.8 (122/130) | 96.3 (235/244) | 93.8 (122/130) |
| Week 89 | 97.5 (233/239) | 95.7 (111/116) | 97.5 (233/239) | 95.7 (111/116) |
| Week 97 | 98.2 (223/227) | 96.2 (101/105) | 98.2 (223/227) | 96.2 (101/105) |
| Week 105 | 97.7 (213/218) | 95.7 (90/94) | 97.7 (213/218) | 95.7 (90/94) |
| Week 113 | 97.6 (200/205) | 95.2 (80/84) | 97.6 (200/205) | 95.2 (80/84) |
| Week 121 | 97.2 (176/181) | 92.6 (63/68) | 97.2 (176/181) | 92.6 (63/68) |
| Week 129 | 98.1 (154/157) | 93.0 (53/57) | 98.1 (154/157) | 93.0 (53/57) |
| Week 137 | 97.8 (135/138) | 93.3 (42/45) | 97.8 (135/138) | 93.3 (42/45) |
| Week 145 | 96.6 (113/117) | 92.5 (37/40) | 96.6 (113/117) | 92.5 (37/40) |
| Week 161 | 97.2 (70/72) | 94.7 (18/19) | 97.2 (70/72) | 94.7 (18/19) |
| Week 177 | 100 (21/21) | 83.3 (5/6) | 100 (21/21) | 83.3 (5/6) |

Data are % (n/N).

## Table S2. Baseline pain levels in Asian vs non-Asian ethnic groups

|  | **Rezvilutamide plus ADT** | | **Bicalutamide plus ADT** | |
| --- | --- | --- | --- | --- |
|  | **Asian (N=295)** | **Non-Asian (N=31)*** | **Asian (N=296)** | **Non-Asian (N=32)** |
| No pain | 187 (63.4) | 16 (51.6) | 192 (64.9) | 14 (43.8) |
| Mild | 48 (16.3) | 6 (19.4) | 44 (14.9) | 6 (18.8) |
| Moderate | 53 (18.0) | 9 (29.0) | 48 (16.2) | 10 (31.3) |
| Severe | 7 (2.4) | 0 | 12 (4.1) | 2 (6.3) |

Data are n (%). *In the non-Asian cohort, one patient from the rezvilutamide group was identified as Hispanic or Latino, while all other non-Asian patients were classified as Non-Hispanic or Latino.

## Table S3. Baseline PSA levels or metastatic burden as stratified by baseline pain severity

|  | **Rezvilutamide plus ADT** | | | | **Bicalutamide plus ADT** | | | |
| --- | --- | --- | --- | --- | --- | --- | --- | --- |
|  | **No pain (N=203)** | **Mild (N=54)** | **Moderate (N=62)** | **Severe (N=7)** | **No pain (N=206)** | **Mild (N=50)** | **Moderate (N=58)** | **Severe (N=14)** |
| Baseline PSA | 71.2 (12.2–293.0) | 55.1 (12.5–185.4) | 130.2 (31.9–566.2) | 100 (57.4–741.2) | 31.9 (6.4–167.5) | 99.3 (10.1–278.3) | 81.5 (10.7–531.7) | 237.2 (40.1–1000) |
| Visceral metastases (excluding lymph nodes) | | | | | | | | |
| Yes | 34 (16.7) | 17 (31.5) | 12 (19.4) | 1 (14.3) | 44 (21.4) | 7 (14.0) | 14 (24.1) | 5 (35.7) |
| No | 169 (83.3) | 37 (68.5) | 50 (80.6) | 6 (85.7) | 162 (78.6) | 43 (86.0) | 44 (75.9) | 9 (64.3) |
| Number of bone lesions | | | | | | | | |
| N≤10 | 62 (30.5) | 18 (33.3) | 22 (35.5) | 0 | 69 (33.5) | 12 (24.0) | 17 (29.3) | 5 (35.7) |
| 10<N≤20 | 45 (22.2) | 14 (25.9) | 11 (17.7) | 2 (28.6) | 49 (23.8) | 11 (22.0) | 10 (17.2) | 2 (14.3) |
| N>20 | 96 (47.3) | 22 (40.7) | 29 (46.8) | 5 (71.4) | 88 (42.7) | 27 (54.0) | 31 (53.4) | 7 (50.0) |
| Extent of metastatic disease | | | | | | | | |
| Bone only | 76 (37.4) | 21 (38.9) | 22 (35.5) | 4 (57.1) | 86 (41.7) | 17 (34.0) | 21 (36.2) | 3 (21.4) |
| Soft tissue only | 6 (3.0) | 1 (1.9) | 1 (1.6) | 0 | 1 (0.5) | 1 (2.0) | 0 | 0 |
| Bone and soft tissue | 121 (59.6) | 32 (59.3) | 39 (62.9) | 3 (42.9) | 119 (57.8) | 32 (64.0) | 37 (63.8) | 11 (78.6) |

Data are median (IQR) or n (%). Metastasis were assessed by investigators.

## Table S4. Between-group comparison of PSA progression, rPFS, and OS as stratified by baseline pain severity

|  | **25th percentile (95% CI)*, months** | | **HR (95% CI)** | **P value** |
| --- | --- | --- | --- | --- |
|  | **Rezvilutamide plus ADT (n=326)** | **Bicalutamide plus ADT (n=328)** |  |  |
| Time to PSA progression | | | | |
| No pain | 25.8 (18.4–31.3) | 5.6 (4.6–6.5) | 0.22 (0.16–0.30) | <0.001 |
| Mild | 18.4 (7.4–31.3) | 6.5 (3.7–8.3) | 0.24 (0.14–0.43) | <0.001 |
| Moderate | 18.5 (8.3–NE) | 5.5 (3.8–6.5) | 0.24 (0.14–0.42) | <0.001 |
| Severe | 14.7 (4.6–NE) | 6.4 (4.6–10.2) | 0.26 (0.06–1.17) | 0.057 |
| rPFS | | | | |
| No pain | 25.8 (18.5–NE) | 10.7 (7.4–11.1) | 0.41 (0.29–0.58) | <0.001 |
| Mild | 18.4 (8.3–25.8) | 14.7 (7.3–29.5) | 0.85 (0.43–1.69) | 0.639 |
| Moderate | 11.3 (10.6–NE) | 8.9 (5.7–11.0) | 0.35 (0.20–0.61) | <0.001 |
| Severe | 6.7 (1.2–NE) | 16.0 (7.4–23.5) | 0.89 (0.21–3.79) | 0.875 |
| OS | | | | |
| No pain | 33.3 (25.4–NE) | 22.0 (20.5–25.2) | 0.57 (0.40–0.82) | 0.002 |
| Mild | 29.5 (14.3–NE) | 28.1 (11.5–36.2) | 0.76 (0.36–1.60) | 0.468 |
| Moderate | 26.4 (12.9–NE) | 15.7 (9.4–17.3) | 0.41 (0.23–0.73) | 0.002 |
| Severe | 6.7 (1.2–NE) | 18.0 (11.0–NE) | 1.64 (0.36–7.35) | 0.516 |

*The 25th percentiles were reported here because the majority of the median values had not been reached.

## Table S5. The superiorities of rezvilutamide plus ADT over bicalutamide plus ADT in pain progression in Asians and non-Asians

|  | **25th percentile (95% CI)*, months** | | **HR (95% CI)** | **P value** |
| --- | --- | --- | --- | --- |
|  | **Rezvilutamide plus ADT (n=326)**** | **Bicalutamide plus ADT (n=328)**** |  |  |
| Time to worst pain progression | | | | |
| Asians | 9.2 (7.3–16.7) | 6.4 (5.5–8.3) | 0.73 (0.55–0.95) | 0.018 |
| Non-Asians | 10.2 (3.8–NE) | 15.1 (1.9–NE) | 1.11 (0.42–2.94) | 0.831 |
| Time to pain interference progression | | | | |
| Asians | 20.3 (14.7–31.4) | 10.2 (7.4–11.7) | 0.68 (0.50–0.93) | 0.014 |
| Non-Asians | 10.2 (3.8–NE) | 7.4 (1.0–NE) | 0.79 (0.31–1.98) | 0.607 |
| Time to average pain progression | | | | |
| Asians | 25.8 (14.9–NE) | 11.7 (8.2–22.1) | 0.77 (0.56–1.07) | 0.115 |
| Non-Asians | 14.7 (4.7–NE) | 12.8 (3.8–NE) | 1.01 (0.35–2.94) | 0.984 |

*The 25th percentiles were reported here because the majority of the median values had not been reached. **In the rezvilutamide plus ADT group, there were 295 Asians and 31 non-Asians, and in the bicalutamide plus ADT group, there were 296 Asians and 32 non-Asians.

## Figure S1


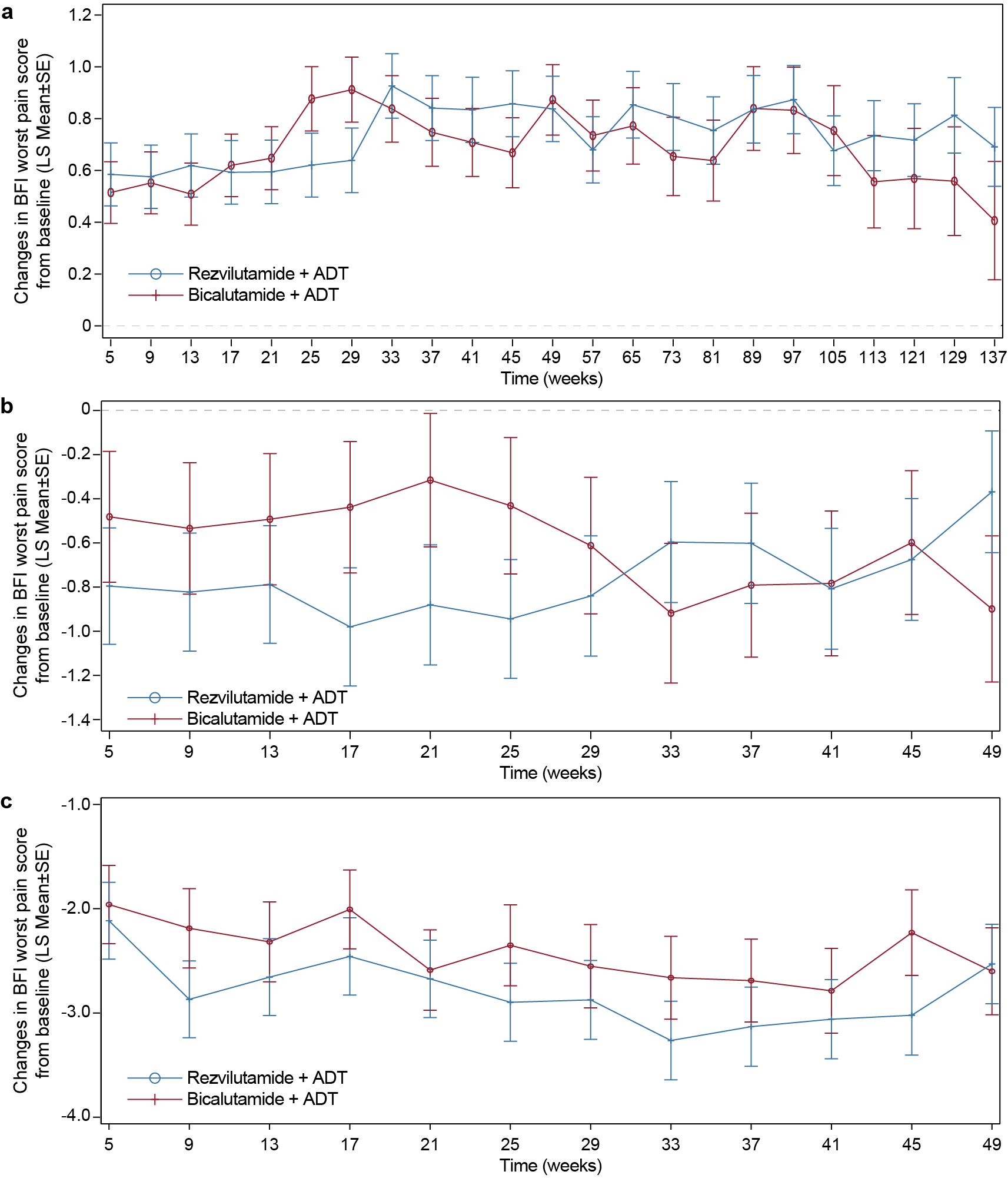


**Figure S1. Changes in worst pain score in past 24 hours from baseline as stratified by baseline pain severity**

(**a**) Patients with no pain at baseline. (**b**) Patients with mild pain at baseline. (**c**) Patients with moderate pain at baseline.

Pain was assessed by use of the Brief Pain Inventory-Short Form (BPI-SF) questionnaire. The BPI-SF comprises a total of 15 items measuring 2 domains: pain severity and pain interference. BPI-SF questionnaire evaluated the severity of pain and the impact of pain on daily function in the past 24 hour on a scale from 0 to 10, with 0 representing “no pain” or “no interference” and 10 being “the worst imaginable pain” or “complete interference”, with higher scores indicating worser.

## Figure S2


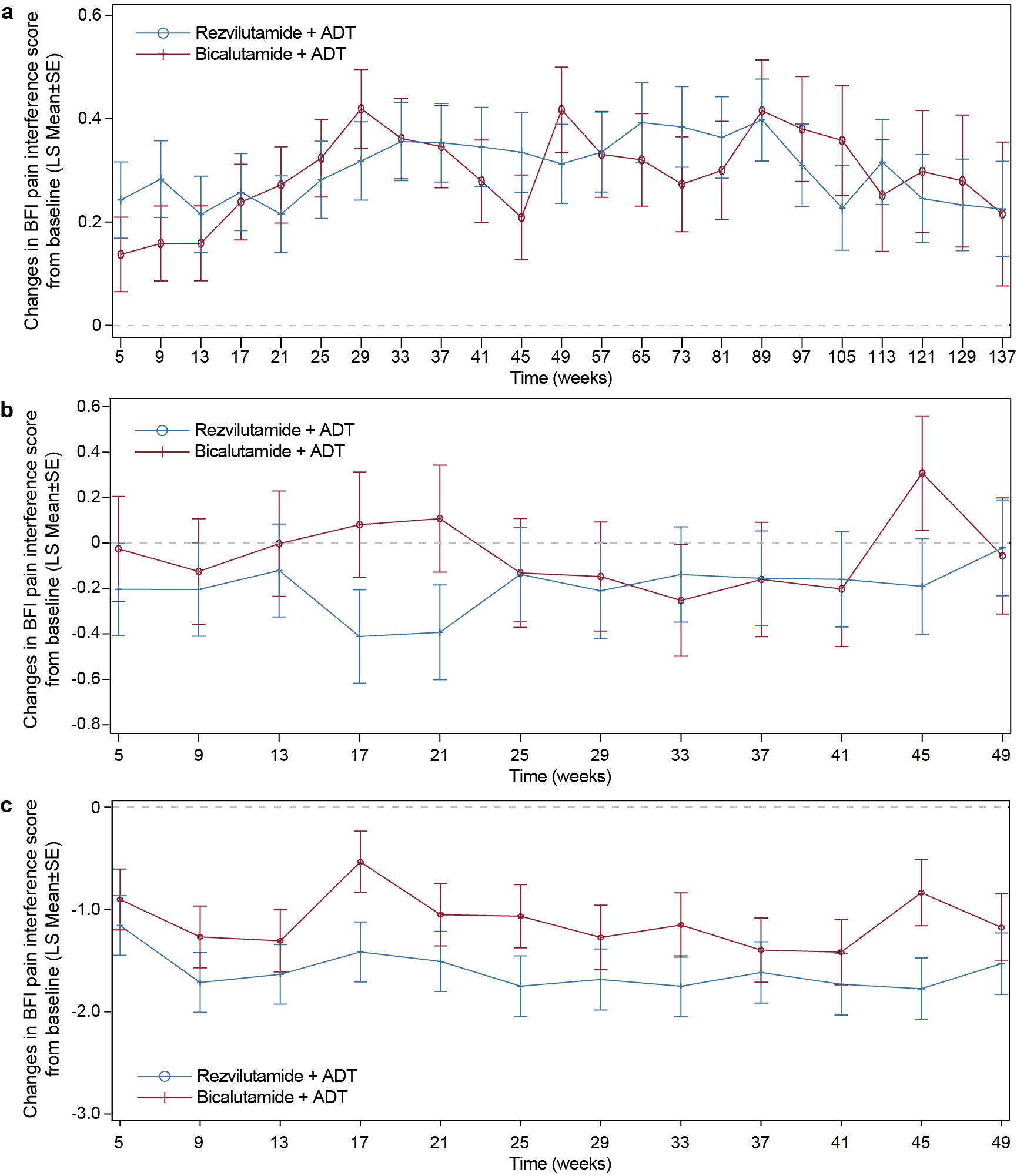


**Figure S2. Changes in pain interference score from baseline as stratified by baseline pain severity**

(**a**) Patients with no pain at baseline. (**b**) Patients with mild pain at baseline. (**c**) Patients with moderate pain at baseline.

Pain was assessed by use of the Brief Pain Inventory-Short Form (BPI-SF) questionnaire. The BPI-SF comprises a total of 15 items measuring 2 domains: pain severity and pain interference. BPI-SF questionnaire evaluated the severity of pain and the impact of pain on daily function in the past 24 hour on a scale from 0 to 10, with 0 representing “no pain” or “no interference” and 10 being “the worst imaginable pain” or “complete interference”, with higher scores indicating worser.

## Figure S3


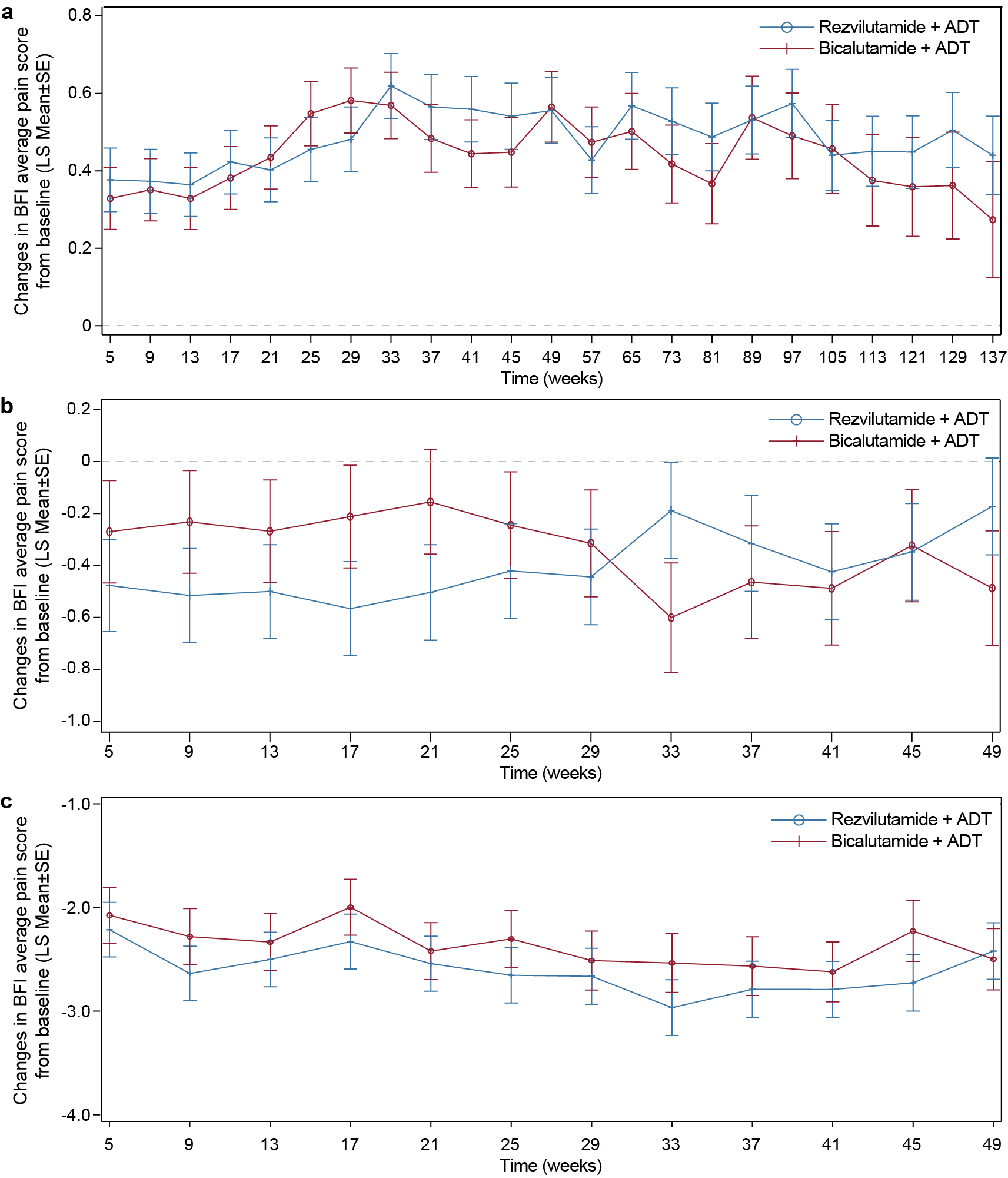


**Figure S3. Changes in average pain score from baseline as stratified by baseline pain severity**

(**a**) Patients with no pain at baseline. (**b**) Patients with mild pain at baseline. (**c**) Patients with moderate pain at baseline.

Pain was assessed by use of the Brief Pain Inventory-Short Form (BPI-SF) questionnaire. The BPI-SF comprises a total of 15 items measuring 2 domains: pain severity and pain interference. BPI-SF questionnaire evaluated the severity of pain and the impact of pain on daily function in the past 24 hour on a scale from 0 to 10, with 0 representing “no pain” or “no interference” and 10 being “the worst imaginable pain” or “complete interference”, with higher scores indicating worser.

## Figure S4


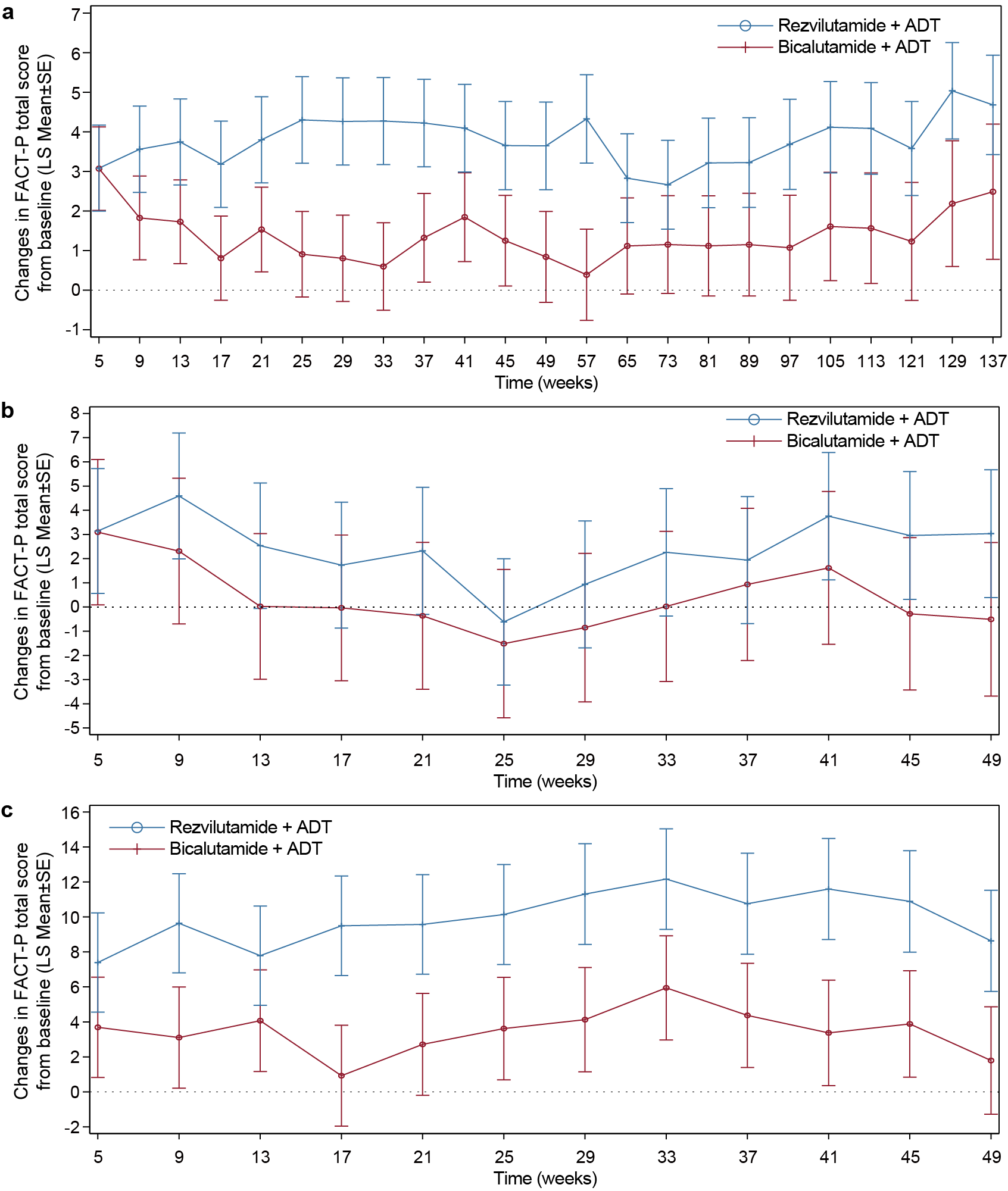


**Figure S4. Change in FACT-P total score from baseline as stratified by baseline pain severity**

(**a**) Patients with no pain at baseline. (**b**) Patients with mild pain at baseline. (**c**) Patients with moderate pain at baseline.

Pain was assessed by use of the Brief Pain Inventory-Short Form (BPI-SF) questionnaire. The BPI-SF comprises a total of 15 items measuring 2 domains: pain severity and pain interference. BPI-SF questionnaire evaluated the severity of pain and the impact of pain on daily function in the past 24 hour on a scale from 0 to 10, with 0 representing “no pain” or “no interference” and 10 being “the worst imaginable pain” or “complete interference”, with higher scores indicating worser.

Functional status was assessed by use of the Functional Assessment of Cancer Therapy-Prostate (FACT-P) questionnaire. The FACT-P questionnaire consists physical wellbeing, social and family wellbeing, emotional wellbeing, functional wellbeing domains, and prostate cancer-specific domain, which comprise the FACT-P total scale (0-156), the FACT-P general function status subscale (FACT-G, consisting of physical wellbeing, social and family wellbeing, emotional wellbeing, and functional wellbeing; 0-108), and the trial outcome index (consisting physical wellbeing, functional wellbeing, and prostate cancer-specific domain), with higher scores indicating better.
