# Supplementary material for: Patient-reported outcomes of rezvilutamide versus bicalutamide in combination with androgen deprivation therapy in high-volume metastatic hormone-sensitive prostate cancer patients (CHART): a randomized, phase 3 study
Source: Signal Transduct Target Ther. 2024 Dec 18;9:351. doi: 10.1038/s41392-024-02064-z (PMC11652652; doi:10.1038/s41392-024-02064-z)
Supplement: Supplementary file 3 — Plagiarism checking report [file 41392_2024_2064_MOESM3_ESM.pdf]

No.2550 -- 13Nov(1).docx

WORD COUNT	5666	TIME SUBMITTED	13-NOV-2024 11:27AM
		PAPER ID	112901875

Article

Patient-reported outcomes of rezvilutamide versus bicalutamide in combination with androgen deprivation therapy in high-volume metastatic hormone-sensitive prostate cancer patients (CHART): a randomized, phase 3 study

Running title: Rezvilutamide plus ADT in mHSPC

Abstract

The randomized phase 3 CHART trial (NCT03520478) revealed that rezvilutamide (REZ) plus androgen deprivation therapy (ADT) in high-volume, metastatic, hormone-sensitive prostate cancer (mHSPC) significantly enhanced radiographic progression-free and overall survival than bicalutamide (Bic)-ADT. Accordingly, we examined patient-reported outcomes (PROs) results, which were exploratory endpoints in the CHART trial. The patients were randomly allocated to receive REZ-ADT or Bic-ADT in a 1:1 ratio. The PROs were evaluated with the Brief Pain Inventory-Short Form (BPI-SF) and the Functional Assessment of Cancer Therapy-Prostate (FACT-P) questionnaires. Both study groups displayed comparable baseline pain scores and functional status. Patients administered REZ-ADT had an extended time to progression of worst pain intensity in comparison to those treated with Bic-ADT (25th percentile, 9.2 [95% CI 7.4–16.6] vs. 6.4 months [95% CI 5.5–8.3]; HR 0.75 [95% CI 0.57–0.97]; p = 0.026). Similarly, patients received REZ-ADT exhibited a delayed time to progression of pain interference in comparison to those receiving Bic-ADT (25th percentile, 20.2 [95% CI 12.9–31.3] vs. 10.2 months [95% CI 7.4–11.1]; HR 0.70 [95% CI 0.52–0.93]; p = 0.015). Additionally, the REZ-ADT group demonstrated a prolonged delay in the deterioration of the total score on the FACT-P questionnaire (25th percentile, 12.8 [95% CI 7.4–20.3] vs. 6.0 months [95% CI 4.6–9.2]; HR 0.66 [95% CI 0.50–0.86]; p = 0.002), as well as most of the FACT-P subscale scores, in comparison to the Bic-ADT group. In conclusion, REZ-ADT was superior to Bic-ADT regarding the pain alleviation and enhancement of functional scales for high-volume mHSPC.

Keywords: rezvilutamide plus ADT, metastatic hormone-sensitive prostate cancer, patient-reported outcomes, pain, functional status

Introduction

Prostate cancer is among the prevailing reasons for cancer-linked morbidity and death in men worldwide, with its incidence rising as populations age.[1] Metastatic hormone-sensitive prostate cancer (mHSPC) is defined by a particularly aggressive disease type, where the tumor spreads beyond the prostate gland, commonly to the bones and lymph nodes, leading to challenging complications and reduced survival. Patients with mHSPC experience a high morbidity rate and poor clinical outcomes, and this disease severely impacts their quality of life (QoL), with common symptoms including bone pain, fatigue, urinary issues, and psychosocial distress.[2] This underscores the need for developing treatment approaches that not only effectively control disease progression but also enhance or maintain patient-reported outcomes (PROs) in this population.

The androgen receptor axis is crucial in prostate cancer pathology by driving tumor proliferation and growth.[3,4] While androgen-deprivation therapy (ADT), which reduces circulating testosterone levels, has been the cornerstone of treatment, single-agent ADT alone often fails to sustain long-term efficacy, as prostate cancer cells develop adaptive resistance to testosterone suppression. In recent years, the emergence of androgen receptor axis inhibitors, encompassing enzalutamide, abiraterone acetate, and apalutamide, has achieved a significant advancement in the therapeutic landscape for mHSPC. These agents, when used in combination with ADT, have demonstrated considerable survival benefits and have become integral to the standard of care for mHSPC patients.[5-8] The combination of androgen receptor axis inhibitors with ADT more effectively suppresses androgen receptor signaling than ADT alone, offering

new hope for delaying disease progression and improving survival outcomes in mHSPC patients.[9] For high-volume mHSPC patients who exhibit an extensive tumor burden with multiple metastatic sites, these new drugs have shown significant potential in clinical trials, resulting in prolonged progression-free survival (PFS) and overall survival (OS) benefits. Notwithstanding these advancements, some patients experience resistance to existing androgen receptor-targeting therapies,[9] underscoring the need for novel agents that can further enhance clinical outcomes, especially for high-volume cases where the tumor burden is substantial and aggressive. Consequently, researchers have been investigating new-generation androgen receptor inhibitors that may overcome treatment resistance, improve efficacy, reduce toxicity, and maintain or enhance QoL, which are essential needs in managing the disease course of mHSPC patients.

Rezvilutamide (REZ) is a new orally administrated inhibitor of the androgen receptor that specifically targets the androgen receptor axis.[10,11] A preclinical study conducted in mice has indicated that REZ penetrates the blood-brain barrier to a significantly lesser extent compared to enzalutamide, potentially lowering the seizure risk. In metastatic castration-resistant prostate cancer patients, clinical studies have demonstrated Rez's clinical effectiveness and favorable safety profile.[12,13] The CHART trial, a randomized phase 3 study, was performed to ascertain the effectiveness and safety of REZ-ADT in high-volume mHSPC patients compared to bicalutamide (Bic)-ADT. This study manifested that the REZ-ADT significantly enhanced OS (Hazard ratio [HR] 0.58 with 95% confidence interval [CI] 0.44–0.77; $p < 0.0001$) and

radiographic progression-free survival (rPFS; HR 0.44 [95% CI 0.33–0.58]; $p < 0.0001$) in mHSPC patients when compared with Bic-ADT.[10,14] These outcomes resulted in the approval of REZ-ADT in China in 2022 for treating high-volume mHSPC.[11] The CHART trial results underscore the importance of incorporating novel androgen receptor-targeting agents into the treatment paradigm for metastatic prostate cancer, especially given the growing body of evidence that these therapies can enhance clinical outcomes and offer new hope for patients with poor prognosis.

In addition to improving clinical efficacy, PROs have emerged as critical endpoints in the evaluation of treatment approaches for mHSPC. Patients with mHSPC often endure not only the physical burden of the disease but also a spectrum of adverse effects from treatment, such as fatigue, hot flashes, loss of libido, and cognitive changes.[15,16] These side effects, combined with disease-related symptoms, can severely impact the QoL and mental well-being of patients.[17] Furthermore, mHSPC disproportionately affects older adults, who may have additional comorbidities that can exacerbate the impact of both disease symptoms and treatment toxicity. Traditional clinical study endpoints, such as OS and rPFS, while essential for assessing treatment efficacy, often fail to capture the daily QoL concerns of patients.[18] Moreover, PROs, which assess symptom burden, functional status, and overall satisfaction with therapy from the perspectives of patients, offer valuable insights into how treatments impact daily functioning and overall well-being, providing a more comprehensive view of therapeutic benefits.[19,20] Incorporating PROs into the study endpoints of clinical trials conducted in mHSPC patients allows for patient-centered evaluations and informs

decision-making by simultaneously considering efficacy, safety, and QoL, ultimately aiming to optimize comprehensive care and improve patient satisfaction.[18]

In the CHART trial, PROs were assessed as exploratory endpoints, focusing on pain control and functional assessments in mHSPC patients receiving either REZ-ADT or Bic-ADT. In this report, we present the comparative PRO data from the CHART phase 3 trial, underscoring the importance of evaluating patient-centered outcomes alongside effectiveness and safety in treatment strategies for high-volume mHSPC.

Results

Patients

Between June 28, 2018, and August 6, 2020, 654 patients fulfilling the eligibility criteria were recruited, with 326 and 328 patients allocated to the REZ-ADT and Bic-ADT groups, respectively. Baseline characteristics, including demographics, disease status, prior treatments, pain levels, and functional scores, were comparable across both treatment groups (Table 1).[14]

This exploratory analysis of PROs had a median follow-up period of 29.3 months (interquartile range [IQR]: 21.0–33.3), with a data cutoff date of February 28, 2022. The PRO data beyond 44 treatment cycles were excluded because the Bic-ADT group had a limited number of remaining patients.

Brief Pain Inventory-Short Form (BPI-SF)

Compliance rates for BPI-SF were high throughout the study, with over 90% compliance observed in both treatment groups up to week 161 (Supplementary Table 1). Only 25 patients (3.8%) missed three or more scheduled pain assessments. Baseline assessments showed low pain levels across both groups, with 203 (62%) in the REZ-ADT group and 206 (63%) in the Bic-ADT group reporting no pain at baseline, while 17% and 15%, respectively, reported mild pain (Table 1). Notably, Asian patients generally reported lower baseline pain levels than non-Asian patients (Supplementary Table 2).

Patients treated with REZ-ADT demonstrated an extended time to progression of worst pain intensity, in contrast to those receiving Bic-ADT (median: NR [95% CI NR–NR] vs. NR [95% CI 20.3–NR]; 25th percentile: 9.2 [95% CI 7.4–16.6] vs. 6.4 months [95% CI 5.5–8.3]; HR 0.75 [95% CI 0.57–0.97]; p = 0.026, Fig. 1a). Similarly, the REZ-ADT group experienced an extended time to progression in pain interference (median: NR [95% CI NR–NR] vs. NR [95% CI NR–NR]; 25th percentile: 20.2 [95% CI 12.9–31.3] vs. 10.2 months [95% CI 7.4–11.1]; HR 0.70 [95% CI 0.52–0.93]; p = 0.015, Fig. 1b). Both groups did not achieve the median time to average pain progression; the REZ-ADT group showed 25th percentile values of 25.8 months (95% CI 14.8–31.4), while the Bic-ADT group indicated 11.7 months (95% CI 8.7–22.1; HR 0.79 [95% CI 0.58–1.08]; p = 0.133; Fig. 1c).

Both therapy groups exhibited decreases in worst pain intensity, interference, and

average pain over time, with more pronounced reductions found in the REZ-ADT group at various time intervals (Fig. 2). Analysis of the initial 12 treatment cycles stratified by baseline pain severity revealed that patients reporting moderate baseline pain showed marked improvements across pain metrics, with least-squares mean (LS Mean) reductions ranging from –1.96 to –3.26 for worst pain intensity, –0.54 to –1.78 for pain interference, and –1.00 to –1.97 for average pain in both groups. The REZ-ADT group frequently experienced a greater enhancement of pain relief than the Bic-ADT cohort (Supplementary Figs. 1–3). Meanwhile, patients with no or mild baseline pain maintained stable pain levels throughout the study.

Baseline assessments indicated higher PSA levels and a greater number of bone metastatic lesions among patients experiencing moderate to severe baseline pain compared to those with mild or no pain in both the REZ-ADT and Bic-ADT groups (Supplementary Table 3). Moreover, no obvious correlations were found between pain severity at baseline and the presence of visceral metastases. Across all baseline pain levels, the REZ-ADT combination consistently delayed PSA progression and prolonged rPFS and OS compared to Bic-ADT, with HRs < 1 across most efficacy outcomes, except for OS improvement in the subgroup with severe pain (Supplementary Table 4). This suggests a consistent therapeutic advantage of REZ-ADT, regardless of initial pain severity.

Additional analyses were conducted to examine the effectiveness of Rez-ADT compared to Bic-ADT in delaying the progression of worst pain intensity, interference, and average pain in Asian and non-Asian patient subgroups (Supplementary Table 5).

Due to a smaller sample size of non-Asian patients, findings for this subgroup should be interpreted with caution.

Functional Assessment of Cancer Therapy-Prostate (FACT-P)

The overall compliance with the FACT-P questionnaire was strong, with adherence rates above 90% in both treatment groups through week 161 (Supplementary Table 1). Only 25 patients (3.8%) missed three or more scheduled functional assessments. Baseline FACT-P scores indicated that the functional status was similar between the REZ-ADT and Bic-ADT groups (Table 1).

For the FACT-P total score, REZ-ADT had longer 25th percentile time to functional deterioration (12.8 months [95% CI 7.4–20.3] compared to 6.0 months [95% CI 4.6–9.2] with Bic-ADT; HR 0.66 [95% CI 0.50–0.86]; p = 0.002; Fig. 3a, Table 2). Deterioration across nearly all FACT-P subscales was delayed with the administration of REZ-ADT, encompassing delayed deterioration in physical well-being (HR 0.65, 95% CI 0.49–0.86; p = 0.003), emotional well-being (HR 0.68, 95% CI 0.50–0.92; p = 0.013), functional well-being (HR 0.75, 95% CI 0.59–0.95; p = 0.015), the FACT-G general scale (HR 0.69, 95% CI 0.52–0.91; p = 0.008), the prostate cancer subscale (HR 0.74, 95% CI 0.57–0.96; p = 0.022), and the trial outcome index (HR 0.65, 95% CI 0.49–0.86; p = 0.002**;** Table 2). Nonetheless, no difference was found between the groups for the time to deterioration in social/family well-being or the FACT-P pain scale.

Both groups demonstrated improvements from baseline in the FACT-P total score at all time points (LS Means of 0.66–6.77; Fig. 3b), with the REZ-ADT group showing

more pronounced improvements, reflected in between-group LS Mean differences of 0.50–5.20. The score changes in all FACT-P subscales from baseline exhibited similar patterns in the improvement in both groups, along with between-group differences, except in the subscales of social/family well-being and the FACT-P pain scale.

An additional analysis evaluated the relationship between FACT-P total score changes over the initial 12 treatment cycles and baseline pain levels. Results suggested that patients with higher baseline pain reported greater improvements in the FACT-P total scale. While patients with low to no pain at baseline showed minor improvements, those with moderate baseline pain reported substantial improvements, with LS Mean scores of 0.92–12.16 overall assessment time points. This trend was more significant in the REZ-ADT group, consistently exceeding improvements in the Bic-ADT group, with between-group LS Mean differences of 3.70–8.57 (Supplementary Fig. 4). The correlations between FACT-P subscale score improvements and baseline pain severity paralleled those observed in the FACT-P total scale, except for the social/family well-being and FACT-P pain subscales.

Discussion

This exploratory analysis of the phase 3 CHART study indicates that REZ-ADT outperformed Bic-ADT in high-volume mHSPC patients in terms of delaying pain progression and enhancing functional health. Treatment with REZ-ADT demonstrated a delayed progression in worst pain intensity, pain interference, and functional deterioration, as assessed by BPI-SF and FACT-P, respectively. The high patient

adherence observed for PRO measures supports the reliability of these assessments in capturing clinically relevant improvements with REZ-based therapy.

Pain management is fundamental in mHSPC care due to its direct impact on patient QoL.[21,22] Our findings suggest that the REZ-ADT group experienced a significant extension in time to pain progression—specifically, in terms of both worst pain and interference scores compared to the Bic-ADT group. However, differences in average pain progression time between groups were minimal. These results align with findings from the LATITUDE trial, which demonstrated that ADT-abiraterone acetate and prednisone delayed the progression of worst pain intensity and interference compared to ADT-placebos.[23] Conversely, the TITAN and ARCHES studies demonstrated no significant differences between apalutamide-ADT and placebo-ADT or enzalutamide-ADT and placebo-ADT regarding the time to progression of worst pain intensity, pain interference, and average pain progression.[24,25] Variations in trial design and patient features should be considered when comparing these results, underscoring the need for further studies on androgen receptor inhibitors combined with ADT to better understand their effects on pain progression.

The analysis of pain relief from baseline scores from worst pain intensity and interference, and average pain favored REZ-ADT at most assessed intervals, suggesting a stronger and more sustained pain-relief effect in this treatment group. Particularly, patients with greater initial pain severity exhibited more substantial improvements in pain-related measures when treated with REZ-ADT, indicating a potential predictive value of baseline pain levels for clinical response to this therapy. These findings are

consistent with data from the TITAN study, where apalutamide-ADT was also more effective in patients with higher baseline pain severity.[24]

Previous studies have shown that there can be variations in pain reporting across ethnic groups, potentially influenced by both cultural and biological factors. Consistent with earlier findings, our results indicated that Asian patients reported lower baseline pain levels,[26] possibly reflecting cultural tendencies such as stoicism, which may affect pain reporting. In contrast, studies suggested that Black men may report higher pain levels than Caucasians, possibly due to variations in pain tolerance, access to healthcare, and historical disparities in pain management for minority populations.[26] From a biological perspective, differences in androgen receptor gene polymorphisms, tumor pathobiology, and hormone metabolism have been observed across ethnic groups,[26] may also contribute to these observed differences in pain reporting both at baseline and during the androgen receptor inhibitor treatment. Although the CHART trial did not specifically address these factors, understanding these variations is essential for interpreting treatment outcomes in diverse patient groups. Future studies in more demographically varied populations, with subgroup analyses by ethnicity, would provide valuable perspectives into the effectiveness of REZ-ADT for managing pain in mHSPC across different backgrounds.

Our analysis using the FACT-P questionnaire revealed that patients treated with REZ-ADT experienced a longer time to functional deterioration across the total scale and more subscales than those in the Bic-ADT group, indicating a sustained preservation of functional well-being. This result suggests that REZ-ADT supports a

better QoL by delaying declines in overall functioning. Although prior studies have shown positive impacts of androgen receptor inhibitors combined with ADT on functional outcomes,[23-25] our findings add robust evidence to this growing body of research. However, differences between groups in social/family well-being and the FACT-P pain scale were not obvious, warranting further exploration to understand the underlying causes.

The trend of improvement in FACT-P scores from baseline across the total scale and nearly all subscales consistently favored REZ-ADT, underscoring its effectiveness in sustaining functional well-being in high-volume mHSPC patients. Correlation analyses showed that patients with higher baseline pain severity experienced the most notable improvements, suggesting that baseline characteristics like pain severity could guide more individualized treatment strategies.

The progression of pain, PSA progression, and survival outcomes relative to metastatic burden observed in the Bic arm were generally consistent with existing findings,[14,27-32] supporting the representativeness of this study population and the reliability of the findings. Although direct comparisons across studies should be interpreted cautiously, the data here suggest a distinct advantage in both efficacy and PROs for REZ over Bic when used with ADT in high-volume mHSPC. The observed improvement in survival and maintenance or enhancement of QoL in patients treated with REZ-ADT can likely be attributed to several potential mechanisms. The selective inhibition of REZ on androgen receptor pathways likely plays a central role by blocking cancer cell growth, reducing tumor burden, and delaying disease progression, especially

pronounced in bone metastases where pain and functional decline are common.[14] Additionally, REZ may help maintain physical mobility and reduce discomfort linked to bone metastasis by limiting osteoclast-mediated bone breakdown. This inhibition of bone resorption could alleviate complications and support mobility, addressing a significant QoL concern for high bone metastatic burden patients.[33,34] Another possible mechanism involves the influence of REZ on molecules associated with pain, such as nerve growth factor and inflammatory cytokines at metastatic sites,[35] which could lead to pain reduction. Moreover, its regulatory impact on the hypothalamic-pituitary-adrenal axis,[36,37] may reduce stress hormone levels, such as cortisol, contributing to improved mental well-being and, in turn, better physical functioning. Altogether, these mechanisms underscore that the survival benefits of REZ-ADT do not come at the expense of QoL; instead, they may enhance it. These insights enable clinicians to have more comprehensive discussions with patients, offering a treatment choice that prioritizes both extended survival and sustained QoL.

Herein, there are several limitations. The current follow-up duration of 29.3 months may not fully capture the entire spectrum of PROs, particularly in the REZ-ADT group, which exhibited superior anti-tumor efficacy and a more favorable PRO profile compared to the Bic-ADT group, with a substantial proportion of patients still receiving treatment. Therefore, an extended follow-up period will be crucial for assessing the long-term durability of these findings. Additionally, since most participants were Asian, the applicability of our study to broader, ethnically diverse populations might be limited. Future studies and meta-analyses incorporating global data can help assess the

consistency of these results across different demographics, enhancing our understanding of the broader applicability of REZ-ADT for high-volume mHSPC patients worldwide.

To conclude, the CHART study establishes the benefits of REZ over Bic when combined with ADT for managing pain and maintaining functional status in high-volume mHSPC. Together with its demonstrated anti-tumor efficacy and safety, these findings reinforce REZ-ADT as a promising treatment option and a potential new standard of therapy for these patients.

Methods

Study Design and Patient Population

In 72 hospitals across China, Poland, the Czech Republic, and Bulgaria, a multinational, randomized, open-label, active-controlled phase 3 CHART study (NCT03520478) was performed. Eligibility criteria were men aged 18 years or above, having histologically or cytologically verified high-volume prostate adenocarcinoma. High-volume disease was characterized by the existence of four or more bone lesions identified using a [99Tc] bone scan, with a minimum of one lesion situated outside the pelvis or vertebral column or by the presence of visceral metastases (excluding lymph node involvement) verified via CT or MRI. Eligible patients needed to have an Eastern Cooperative Oncology Group (ECOG) performance status of 0 or 1 and sufficient organ function. Prior treatment with ADT was permitted if administered no more than three months before study entry, provided there was no evidence of significant radiographic

or clinical progression of prostate-specific antigen levels. Patients exhibiting neuroendocrine differentiation or small-cell characteristics, or those with a history of chemotherapy or localized treatment, were excluded. Nevertheless, patients who had received a single course of palliative radiotherapy, transurethral resection of the prostate, or surgical interventions for metastatic symptoms were eligible, provided these interventions were completed at least four weeks prior to treatment initiation and all treatment-related adverse events resolved to grade 1 or 0, as per the Common Terminology Criteria for Adverse Events version 4.03.

The study protocol and its amendments were approved by independent ethics review committee at each involved site. The trial adhered to the Declaration of Helsinki and the International Council for Harmonization Good Clinical Practice guidelines. All participants provided informed consent.

Treatment and Assessments

Eligible patients were randomly allocated to receive ADT (utilizing a luteinizing hormone-releasing hormone agonist or antagonist or bilateral orchiectomy) combined with either REZ (240 mg) or Bic (50 mg) in a 1:1 ratio, received orally once daily in 28-day therapy cycles. Randomization was stratified according to ECOG performance status (0 or 1) and the existence of visceral metastases (yes or no). Treatment continued until disease progression, unacceptable toxicity, withdrawal of consent, or decision by the investigator. Dose interruptions and reductions for REZ were allowed, but dose

interruptions for Bic were allowed to manage toxicity, but dose reductions were not permitted. Pain management was guided by the World Health Organization's analgesic ladder and local pain management protocols in China.[38-41]

The PROs were evaluated with BPI-SF and the FACT-P version 4 questionnaires. The BPI-SF has 15 items that evaluate two main domains: pain severity and pain interference.[42,43] Pain severity and its effect on everyday activities were rated on a scale from 0 to 10, with 0 denoting "no pain" or "no interference" and 10 signifying "the worst imaginable pain" or "complete interference." Higher scores indicate worse pain experiences. The FACT-P questionnaire encompasses domains of physical, social, family, emotional, and functional well-being, and a prostate cancer-specific domain. It comprises the total FACT-P scale (0–156), the FACT-P subscale for general functional status (FACT-G, which includes physical, social and family, emotional, and functional well-being; 0–108), and the trial outcome index (which includes physical and functional well-being, and the prostate cancer-specific domain), with higher scores reflecting better outcomes.[44-46] At baseline, the questionnaires were administered, as well as at the commencement of each cycle from cycles 2 to 12, bi-cyclically during cycles 13 to 36, and every four cycles afterward, continuing until 30 days following the last treatment. Patient-reported data were not collected throughout long-term survival follow-ups.

Outcomes

The co-primary endpoints of the CHART trial encompassed independent review committee-evaluated rPFS and OS, with data previously published. PROs were

exploratory endpoints evaluated using the BPI-SF and the FACT-P (version 4).

The time to the worst pain intensity progression was defined as the interval from randomization to the first occurrence of a 2-point or greater rise in pain intensity, as assessed by item 3 of the BPI-SF. The time to pain interference progression was calculated as the period from randomization to the first occurrence of an increase of at least half a standard deviation from baseline scores on the combined scale of items 9A-G of the BPI-SF. The time to average pain progression was defined as the period from randomization to the first reported rise of 2 points or more in average pain compared to baseline, determined by the average of items 3-6 of the BPI-SF. Deterioration thresholds for the FACT-G general scale, trial outcome index, FACT-P total scale, and FACT-P pain scale were established at 9 points, 9 points, 10 points, and 2 points, respectively; for other FACT-P scales; the threshold for deterioration was set at 3 points. Confirmatory evaluations for these progressions were required at subsequent assessments conducted at least four weeks later. In cases where the last assessment indicated progression but there was no confirmed progression, the last assessment was treated as an event.

Statistical Analyses

Details regarding sample size assumptions have been previously reported. PROs were analyzed in the intent-to-treat population, including all randomized patients.

Compliance, BPI-SF scale scores, and FACT-P scale scores were summarized descriptively. The Kaplan-Meier methodology was employed to calculate the median

time to deterioration for each therapy group, with 95% CIs for median values estimated with the Brookmeyer-Crowley method. Between-group differences in time-to-event parameters were analyzed utilizing a stratified log-rank test. A stratified Cox proportional hazards model was emloyed to derive the HR and their corresponding 95% CIs. In cases where median values could not be ascertained, comparisons were made using the 25th percentiles. The censoring date for time to PRO progression or deterioration of the BPI-SF and FACT-P was defined as the date of the last BPI-SF assessment if no pain progression was noted or the date of randomization if there was no baseline or post-baseline disease assessment.

Alterations from baseline in the BPI-SF and FACT-P scales were analyzed employing a linear Mixed Model Repeated Measures model approach, employing Restricted Maximum Likelihood estimation. The model incorporated the baseline values, treatment groups, stratification factors, visits, and treatment-by-visit interactions, treating patients as random impacts. An unstructured covariance matrix was utilized, with a fallback to compound symmetry covariance structures if convergence issues arose. The Kenward-Roger approximation was employed to investigate the freedom degrees. The LS Mean and the differences between groups in LS Means, along with their corresponding 95% CIs, were calculated. The model included visits with at least 30 non-missing values in each treatment group. All statistical analyses were conducted utilizing SAS software (v. 9.4).

Data availability

Available upon a reasonable request from the corresponding author.

Acknowledgments

We extend our appreciation to the patients, their families, the investigators, the site workers, and the trial teams. Medical writing support was offered by Tengfei Zhang (PhD, medical writer at Jiangsu Hengrui Pharmaceuticals Co., Ltd) in accordance with Good Publication Practice Guidelines.

Author contributions

HW, DY, JL, and WW performed the conception and design of the study. All authors participated in the data collection. XY conducted the statistical analysis. All authors performed data interpretation and manuscript writing and reviewed and approved the submission.

Competing interests

XY, JL, and WW are employees of Jiangsu Hengrui Pharmaceuticals Co., Ltd. The remaining co-authors declare no competing interests.

Funding

Funded by Jiangsu Hengrui Pharmaceuticals Co., Ltd.

Figure legends

Figure 1. Kaplan-Meier curves for time to pain progression assessed by BPI-SF

(**a**) time to worst pain progression. (**b**) time to pain interference progression. (**c**) time to average pain progression.

Figure 2. Changes in pain from baseline assessed by BPI-SF

(**a**) Worst pain in past 24 hours. (**b**) Pain interference. (**c**) Average pain.

Figure 3. FACT-P total score

(a) Kaplan-Meier curve for time to functional status deterioration assessed by FACT-P total score. (**b**) Change from baseline in FACT-P total score.

References

1 Bray, F. et al. Global cancer statistics 2022: GLOBOCAN estimates of incidence and mortality worldwide for 36 cancers in 185 countries. CA Cancer J Clin. 74, 229-263 (2024).

2 Sandhu, S. et al. Prostate cancer. Lancet. 398, 1075-1090 (2021).

3 Dai, C., Dehm, S. M. & Sharifi, N. Targeting the Androgen Signaling Axis in Prostate Cancer. J Clin Oncol. 41, 4267-4278 (2023).

4 Dai, C., Heemers, H. & Sharifi, N. Androgen Signaling in Prostate Cancer. Cold Spring Harb Perspect Med. 7 (2017).

5 Fizazi, K. et al. Abiraterone plus prednisone in metastatic, castration-sensitive prostate cancer. N Eng J Med. 377, 352-360 (2017).

6 Chi, K. N. et al. Apalutamide for metastatic, castration-sensitive prostate cancer. N Eng J Med. 381, 13-24 (2019).

7 Davis, I. D. et al. Enzalutamide with standard first-line therapy in metastatic prostate cancer. N Eng J Med. 381, 121-131 (2019).

8 Armstrong, A. J. et al. ARCHES: a randomized, phase III study of androgen deprivation therapy with enzalutamide or placebo in men with metastatic hormone-sensitive prostate cancer. J Clin Oncol. 37, 2974-2986 (2019).

9 Hussain, M. et al. Metastatic Hormone-Sensitive Prostate Cancer and Combination Treatment Outcomes: A Review. JAMA Oncol. 10, 807-820 (2024).

10 Singh, K. et al. Rezvilutamide for metastatic hormone-sensitive prostate cancer. Lancet Oncol. 23, e490 (2022).

11 Keam, S. J. Rezvilutamide: First Approval. Drugs. 83, 189-193 (2023).

12 Qin, X. et al. Activity and safety of SHR3680, a novel antiandrogen, in patients with metastatic castration-resistant prostate cancer: a phase I/II trial. BMC Med. 20, 84 (2022).

13 Qin, X. et al. SHR3680, a novel antiandrogen, for the treatment of metastatic castration-resistant prostate cancer (mCRPC): A phase I/II study. Journal of Clinical Oncology. 38, 90-90 (2020).

14 Gu, W. et al. Rezvilutamide versus bicalutamide in combination with androgen-deprivation therapy in patients with high-volume, metastatic, hormone-sensitive prostate cancer (CHART): a randomised, open-label, phase 3 trial. Lancet Oncol. 23, 1249-1260 (2022).

15 Penson, D. F. & Litwin, M. S. The physical burden of prostate cancer. Urol Clin North Am. 30, 305-313 (2003).

16 Baden, M. et al. Pain, fatigue and depression symptom cluster in survivors of prostate cancer. Support Care Cancer. 28, 4813-4824 (2020).

17 Rhee, H. et al. Adverse effects of androgen-deprivation therapy in prostate cancer and their management. BJU Int. 115 Suppl 5, 3-13 (2015).

18 Efficace, F. et al. Overcoming barriers to the implementation of patient-reported outcomes in cancer clinical trials: the PROMOTION Registry. Health Qual Life Outcomes. 12, 86 (2014).

19 Nipp, R. D. et al. The relationship between physical and psychological symptoms and health care utilization in hospitalized patients with advanced cancer. Cancer. 123, 4720-4727 (2017).

20 Basch, E. et al. Symptom Monitoring With Patient-Reported Outcomes During Routine Cancer Treatment: A Randomized Controlled Trial. J Clin Oncol. 34, 557-565 (2016).

21 Bader, P. et al. Prostate cancer pain management: EAU guidelines on pain management. World J Urol. 30, 677-686 (2012).

22 Thompson, J. C., Wood, J. & Feuer, D. Prostate cancer: palliative care and pain relief. Br Med Bull. 83, 341-354 (2007).

23 Chi, K. N. et al. Patient-reported outcomes following abiraterone acetate plus prednisone added to androgen deprivation therapy in patients with newly diagnosed metastatic castration-naive prostate cancer (LATITUDE): an international, randomised phase 3 trial. Lancet Oncol. 19, 194-206 (2018).

24 Agarwal, N. et al. Health-related quality of life after apalutamide treatment in patients with metastatic castration-sensitive prostate cancer (TITAN): a randomised, placebo-controlled, phase 3 study. Lancet Oncol. 20, 1518-1530 (2019).

25 Stenzl, A. et al. Effect of Enzalutamide plus Androgen Deprivation Therapy on Health-related Quality of Life in Patients with Metastatic Hormone-sensitive Prostate Cancer: An Analysis of the ARCHES Randomised, Placebo-controlled, Phase 3 Study. Eur Urol. 78, 603-614 (2020).

26 Anderson, K. O., Green, C. R. & Payne, R. Racial and ethnic disparities in pain:

causes and consequences of unequal care. J Pain. 10, 1187-1204 (2009).

27 Akaza, H. et al. Combined androgen blockade with bicalutamide for advanced prostate cancer: long-term follow-up of a phase 3, double-blind, randomized study for survival. Cancer. 115, 3437-3445 (2009).

28 Davis, I. D. et al. Enzalutamide with Standard First-Line Therapy in Metastatic Prostate Cancer. N Engl J Med. 381, 121-131 (2019).

29 Shore, N. D. et al. Efficacy and safety of enzalutamide versus bicalutamide for patients with metastatic prostate cancer (TERRAIN): a randomised, double-blind, phase 2 study. Lancet Oncol. 17, 153-163 (2016).

30 Vaishampayan, U. N. et al. Clinical Efficacy of Enzalutamide vs Bicalutamide Combined With Androgen Deprivation Therapy in Men With Metastatic Hormone-Sensitive Prostate Cancer: A Randomized Clinical Trial. JAMA Netw Open. 4, e2034633 (2021).

31 Penson, D. F. et al. Enzalutamide Versus Bicalutamide in Castration-Resistant Prostate Cancer: The STRIVE Trial. J Clin Oncol. 34, 2098-2106 (2016).

32 Ueda, T. et al. Abiraterone acetate versus bicalutamide in combination with gonadotropin releasing hormone antagonist therapy for high risk metastatic hormone sensitive prostate cancer. Sci Rep. 11, 10094 (2021).

33 Logothetis, C. J. & Lin, S. H. Osteoblasts in prostate cancer metastasis to bone. Nat Rev Cancer. 5, 21-28 (2005).

34 Roodman, G. D. Mechanisms of bone metastasis. N Engl J Med. 350, 1655-1664 (2004).

35 Di Donato, M. et al. Cross-talk between androgen receptor and nerve growth factor receptor in prostate cancer cells: implications for a new therapeutic approach. Cell Death Discov. 4, 5 (2018).

36 Kiezun, J., Kaminska, B., Jankowski, J. & Dusza, L. Concentrations of the adrenocorticotropic hormone, corticosterone and sex steroid hormones and the expression of the androgen receptor in the pituitary and adrenal glands of male turkeys (Meleagris gallopavo) during growth and development. Gen Comp Endocrinol. 217-218, 62-70 (2015).

37 Zuloaga, D. G., Lafrican, J. J. & Zuloaga, K. L. Androgen regulation of behavioral stress responses and the hypothalamic-pituitary-adrenal axis. Horm Behav. 162, 105528 (2024).

38 Anekar, A. A., Hendrix, J. M. & Cascella, M. in StatPearls (2024).

39 Mestdagh, F., Steyaert, A. & Lavand'homme, P. Cancer Pain Management: A Narrative Review of Current Concepts, Strategies, and Techniques. Curr Oncol. 30, 6838-6858 (2023).

40 National Health Commission of China. Cancer Pain Treatment Guidelines (2018 Edition). Aug 27, 2018. https://www.gov.cn/xinwen/2018-10/02/5327533/files/76920913ea5f4149b064984fe63636ac.docx (Accessed Sep 10, 2024).

41 Chinese Medical Doctor Association Pain Management Subcommittee. Chinese Expert Consensus on Cancer-Related Pain Assessment (2023 Edition). Dec 15, 2023. http://casp.ijournals.cn/ch/reader/download_pdf.aspx?file_no=231201&year_id=2023

&quarter_id=12&flag=1 (Accessed Sep 10, 2024).

42 Cleeland CS. Brief Pain Inventory (Short Form). National Palliative Care Research Center. 1991. http://www.npcrc.org/files/news/briefpain_short.pdf (accessed Jan 24, 2024).

43 Cleeland, C. S. & Ryan, K. M. Pain assessment: global use of the Brief Pain Inventory. Ann Acad Med Singap. 23, 129-138 (1994).

44 FACIT. Functional Assessment of Cancer Therapy-Prostate (version 4). Nov 19, 2007. https://www.facit.org/_files/ugd/626819_bcdd612dbf734297a32172aa2873d7f4.pdf (accessed Jan 24, 2024).

45 Cella, D. et al. Estimating clinically meaningful changes for the Functional Assessment of Cancer Therapy--Prostate: results from a clinical trial of patients with metastatic hormone-refractory prostate cancer. Value Health. 12, 124-129 (2009).

46 Esper, P. et al. Measuring quality of life in men with prostate cancer using the functional assessment of cancer therapy-prostate instrument. Urology. 50, 920-928 (1997).

No.2550 -- 13Nov(1).docx

ORIGINALITY REPORT

23%
SIMILARITY INDEX

PRIMARY SOURCES

1	Ding-Wei Ye, Shusuan Jiang, Hong Luo, Fangjian Zhou et al. "Patient-reported outcomes (PROs) for rezvilutamide versus bicalutamide in combination with androgen-deprivation therapy (ADT) in high-volume, metastatic, hormone-sensitive prostate cancer (mHSPC): An analysis of the CHART randomized, open-label, phase 3 trial.", Journal of Clinical Oncology, 2024<br>Crossref	178 words — 3%
2	Kim N Chi, Andrew Protheroe, Alfredo Rodríguez-Antolín, Gaetano Facchini et al. "Patient-reported outcomes following abiraterone acetate plus prednisone added to androgen deprivation therapy in patients with newly diagnosed metastatic castration-naive prostate cancer (LATITUDE): an international, randomised phase 3 trial", The Lancet Oncology, 2018<br>Crossref	150 words — 3%
3	www.thelancet.com<br>Internet	103 words — 2%
4	Xiaojie Bian, Weijie Gu, Xuepei Zhang, Liping Xie et al. "Correlation of PSA and survival in metastatic hormone-sensitive prostate cancer treated with rezvilutamide plus ADT in the CHART trial", Med, 2024<br>Crossref	100 words — 2%
5	www.mdpi.com	

Internet

60 words — 1%

6 www.frontiersin.org
Internet

54 words — 1%

7 link.springer.com
Internet

47 words — 1%

8 www-thelancet-com.autorpa.ntunhs.edu.tw
Internet

39 words — 1%

9 www.targetedonc.com
Internet

38 words — 1%

10 Weijie Gu, Weiqing Han, Hong Luo, Fangjian Zhou et al. "Rezvilutamide versus bicalutamide in combination with androgen-deprivation therapy in patients with high-volume, metastatic, hormone-sensitive prostate cancer (CHART): a randomised, open-label, phase 3 trial", The Lancet Oncology, 2022
Crossref

32 words — 1%

11 Fred Saad, Antoine Thiery-Vuillemin, Pawel Wiechno, Boris Alekseev et al. "Patient-reported outcomes with olaparib plus abiraterone versus placebo plus abiraterone for metastatic castration-resistant prostate cancer: a randomised, double-blind, phase 2 trial", The Lancet Oncology, 2022
Crossref

29 words — 1%

12 cdr.lib.unc.edu
Internet

26 words — < 1%

13 Ethan Basch, Karen Autio, Charles J Ryan, Peter Mulders et al. "Abiraterone acetate plus prednisone versus prednisone alone in chemotherapy-naive

23 words — < 1%

men with metastatic castration-resistant prostate cancer: patient-reported outcome results of a randomised phase 3 trial", The Lancet Oncology, 2013
Crossref

14	www.nejm.org<br>Internet	22 words — < 1%
15	D. Ye, S. Wang, Z. Wang, N. Xing et al. "1649P Impact of concomitant medications on safety in patients with high-volume metastatic hormone-sensitive prostate cancer (mHSPC) receiving rezvilutamide (Rez) plus androgen-deprivation therapy (ADT): A post-hoc analysis of the randomized phase III CHART trial", Annals of Oncology, 2024<br>Crossref	21 words — < 1%
16	mdedge.ma1.medscape.com<br>Internet	19 words — < 1%
17	Binghe Xu, Min Yan, Fei Ma, Xichun Hu et al. "Pyrotinib plus capecitabine versus lapatinib plus capecitabine for the treatment of HER2-positive metastatic breast cancer (PHOEBE): a multicentre, open-label, randomised, controlled, phase 3 trial", The Lancet Oncology, 2021<br>Crossref	17 words — < 1%
18	D. Cella, C. Ivanescu, S. Holmstrom, C.N. Bui, J. Spalding, K. Fizazi. "Impact of enzalutamide on quality of life in men with metastatic castration-resistant prostate cancer after chemotherapy: additional analyses from the AFFIRM randomized clinical trial", Annals of Oncology, 2015<br>Crossref	16 words — < 1%
19	aacrjournals.org<br>Internet	15 words — < 1%

20 www.nature.com
Internet
15 words — < 1%

21 Joelle El-Amm, Rami Nassabein, Jeanny Aragon-Ching. "Impact of abiraterone on patient-related outcomes in metastatic castration-resistant prostate cancer: current perspectives", Cancer Management and Research, 2017
Crossref
13 words — < 1%

22 wiadlek.pl
Internet
13 words — < 1%

23 Managing Metastatic Prostate Cancer In Your Urological Oncology Practice, 2016.
Crossref
12 words — < 1%

24 Nussbaum, N, D J George, A P Abernethy, C M Dolan, N Oestreicher, S Flanders, and T B Dorff. "Patient experience in the treatment of metastatic castration-resistant prostate cancer: state of the science", Prostate Cancer and Prostatic Diseases, 2016.
Crossref
12 words — < 1%

25 Yi Xu, Wei Hu, Jian Li, Xin Jiang, Ping Shi, Kai Shen, Yu Shen, Lingyu Ma, Yu Cao. "Safety, pharmacokinetics, and pharmacodynamics of SHR7280, an oral gonadotropin-releasing hormone antagonist in healthy premenopausal women", Frontiers in Pharmacology, 2022
Crossref
11 words — < 1%

26 iris.unito.it
Internet
11 words — < 1%

27 assets.researchsquare.com
Internet
10 words — < 1%

28 trepo.tuni.fi
Internet
10 words — < 1%

29 "Management of Advanced Prostate Cancer", Springer Science and Business Media LLC, 2018
Crossref
9 words — < 1%

30 "Recent progress and controversies in the treatment of metastatic hormone-sensitive prostate cancer", Journal of Men's Health, 2024
Crossref
9 words — < 1%

31 bmcmedicine.biomedcentral.com
Internet
9 words — < 1%

32 flore.unifi.it
Internet
9 words — < 1%

33 www.zora.uzh.ch
Internet
9 words — < 1%

34 Jin-Ji Yang, Cheng Huang, Yun Fan, Hongming Pan et al. "Camrelizumab in different PD-L1 expression cohorts of pre-treated advanced or metastatic non-small cell lung cancer: a phase II study", Cancer Immunology, Immunotherapy, 2021
Crossref
8 words — < 1%

35 Pedro Barata, Umang Swami, Neeraj Agarwal. "The addition of apalutamide to ADT in the treatment of metastatic castration-sensitive prostate cancer: safety and efficacy", Expert Review of Anticancer Therapy, 2020
Crossref
8 words — < 1%

36 Stéphane Oudard, Boris Hadaschik, Fred Saad, David Cella et al. "Health-related Quality of Life at the SPARTAN Final Analysis of Apalutamide for Nonmetastatic
8 words — < 1%

Castration-resistant Prostate Cancer Patients Receiving Androgen Deprivation Therapy", European Urology Focus, 2021
Crossref

---

37 for the NCIC CTG PR.3/MRC UK PR07 investigators. "Combined androgen deprivation therapy and radiation therapy for locally advanced prostate cancer: a randomised, phase 3 trial", The Lancet, 20111217/20120106 — 8 words — < 1%
Crossref

---

38 lirias.kuleuven.be — 8 words — < 1%
Internet

---

39 pubmed.ncbi.nlm.nih.gov — 8 words — < 1%
Internet

---

40 worldwidescience.org — 8 words — < 1%
Internet

---

41 www.onclive.com — 8 words — < 1%
Internet

---

42 Karim Fizazi, Gero Kramer, Jean-Christophe Eymard, Cora N Sternberg et al. "Quality of life in patients with metastatic prostate cancer following treatment with cabazitaxel versus abiraterone or enzalutamide (CARD): an analysis of a randomised, multicentre, open-label, phase 4 study", The Lancet Oncology, 2020 — 7 words — < 1%
Crossref

---

43 Marci J Clark, Nimanee Harris, Ingolf Griebsch, Dagmar Kaschinski, Catherine Copley-Merriman. "Patient-reported outcome labeling claims and measurement approach for metastatic castration-resistant prostate cancer treatments in the United States and European Union", Health and Quality of Life Outcomes, 2014 — 7 words — < 1%
Crossref

---

44 Sumedha Chhatre, Alan J. Wein, S. Bruce Malkowicz, Ravishankar Jayadevappa. "Racial differences in well-being and cancer concerns in prostate cancer patients", Journal of Cancer Survivorship, 2011
Crossref
7 words — < 1%

45 Neeraj Agarwal, Kelly McQuarrie, Anders Bjartell, Simon Chowdhury et al. "Health-related quality of life after apalutamide treatment in patients with metastatic castration-sensitive prostate cancer (TITAN): a randomised, placebo-controlled, phase 3 study", The Lancet Oncology, 2019
Crossref
6 words — < 1%

46 Yutong Lu, Jingqi Jiang, Gaoyang Yang, Hui Ding et al. "Comparative effectiveness of multiple androgen receptor signaling inhibitor medicines with androgen deprivation therapy for metastatic hormone-sensitive prostate cancer: a study in the real world", Frontiers in Oncology, 2024
Crossref
6 words — < 1%

EXCLUDE QUOTES	OFF	EXCLUDE SOURCES	OFF
EXCLUDE BIBLIOGRAPHY	ON	EXCLUDE MATCHES	OFF
